# Saliniquinone Derivatives, Saliniquinones G−I and Heraclemycin E, from the Marine Animal-Derived *Nocardiopsis aegyptia* HDN19-252

**DOI:** 10.3390/md19100575

**Published:** 2021-10-14

**Authors:** Luning Zhou, Xuedong Chen, Chunxiao Sun, Yimin Chang, Xiaofei Huang, Tianjiao Zhu, Guojian Zhang, Qian Che, Dehai Li

**Affiliations:** 1 School of Medicine and Pharmacy, Ocean University of China, Qingdao 266003, China; 18895692529@163.com (L.Z.); chenxuedong1206@163.com (X.C.); sunchunxiao93@163.com (C.S.); yiminchang@163.com (Y.C.); hxf17853102898@163.com (X.H.); zhutj@ouc.edu.cn (T.Z.); zhangguojian@ouc.edu.cn (G.Z.); 2 Laboratory for Marine Drugs and Bioproducts of Qingdao National Laboratory for Marine Science and Technology, Qingdao 266237, China

**Keywords:** anthraquinone derivatives, GNPS, *Nocardiopsis aegyptia*, MRCNS

## Abstract

Four new anthraquinone derivatives, namely saliniquinones G−I (**1**–**3**) and heraclemycin E (**4**), were obtained from the Antarctic marine-derived actinomycete *Nocardiopsis aegyptia* HDN19-252, guided by the Global Natural Products Social (GNPS) molecular networking platform. Their structures, including absolute configurations, were elucidated by extensive NMR, MS, and ECD analyses. Compounds **1** and **2** showed promising inhibitory activity against six tested bacterial strains, including methicillin-resistant coagulase-negative *staphylococci* (MRCNS), with MIC values ranging from 3.1 to 12.5 μM.

## 1. Introduction

Saliniquinones are renowned antibiotics featuring a typical anthraquinone-*γ*-pyrone skeleton [1] and a side chain with different substituents, such as methyl and allyl groups. Since being first described in 1956, [2] more than 50 saliniquinone derivatives have been isolated from various genera, mainly *Streptomyces*. As optically active metabolites, most of them featured *R* configuration at C-15, with only six derivatives assigned as having *S* configuration naturally. Saliniquinones show various biological activities, including cytotoxic [3], antimicrobial [4], and DNA synthesis inhibitory effects [5], etc.

**Figure 1:**
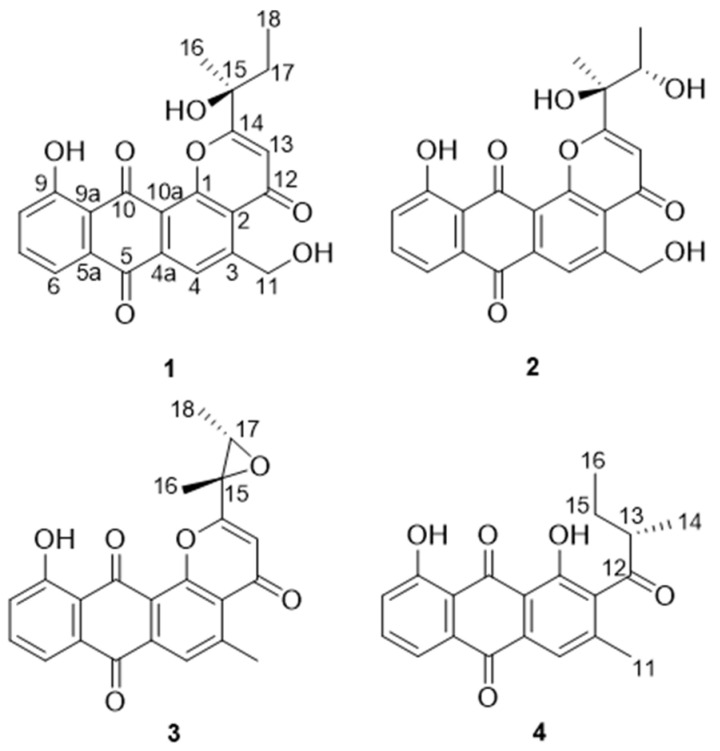
Structures of **1**–**4**.

During our efforts in obtaining new bioactive metabolites from actinomycetes, *Nocardiopsis aegyptia* HDN19-252 was selected for the intriguing UV absorption of EtOAc extract. A comprehensive examination of EtOAc extract using the Global Natural Product Social Molecular Networking (GNPS) platform [6,7], LC-MS-UV, and MarinLit database indicated that the strain *N. aegyptia* HDN19-252 has potential saliniquinone derivatives in the metabolite profile. Moreover, a number of nodes that could not be retrieved in the GNPS platform [6,7] or other databases indicated the existence of new saliniquinone analogues. Followed up by HPLC-UV and LC-MS profiles, three saliniquinone derivatives and one new heraclemycin analogue (Figure 1) were isolated from the crude extract of *N*. *aegyptia* HDN19-252. Among them, **1**–**3** represent the first discovery of saliniquinones produced by *Nocardia* sp., and all of them possess the rare *S* configuration at C-15. Compounds **1**–**4** were evaluated for antibacterial activity against six bacterial strains, including methicillin-resistant coagulase-negative *staphylococci* (MRCNS), *B. subtilis*, *Proteus* sp., *B. cereus*, *Escherichia coli*, and *Mycobacterium phlei.* As a result, compounds **1** and **2** showed broad inhibitory effects. Herein, we report the details of the isolation, structure elucidation, and bioactivities of these compounds.

## 2. Results

**Figure 2:**
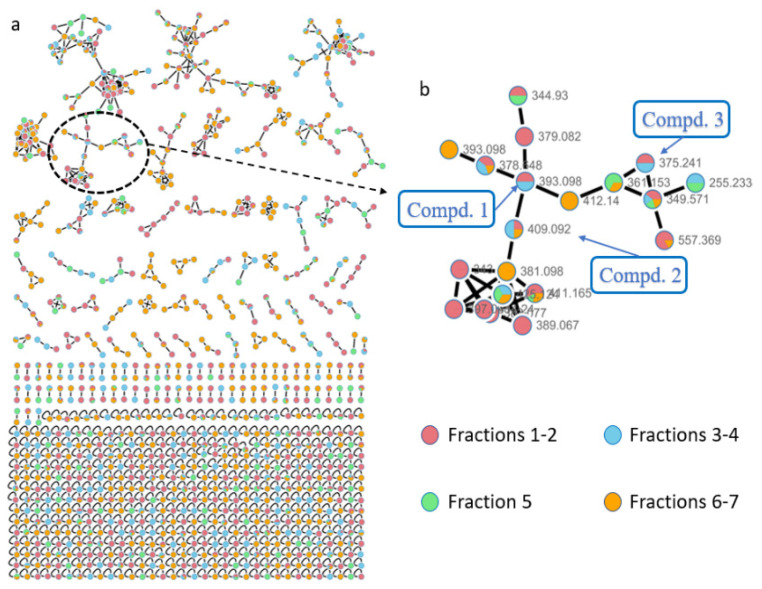
(**a**) Molecular network of all subfractions from *Nocardiopsis aegyptia* HDN19-252; (**b**) cluster corresponding to compounds of the saliniquinone family observed in the molecular network.

The actinomycete strain *N. aegyptia* HDN19-252 was isolated from an unidentified animal (Figure S1) collected form the Antarctic sea. The strain was cultured under static conditions, and the EtOAc extract (10.2 g) was fractionated by vacuum-liquid chromatography (VLC) using an ODS column to obtain seven subfractions, which were further analyzed via the GNPS web platform. A concentrated cluster with nodes attributed to subfractions 1–7 was spotted within the whole molecular network (Figure 2a). Combining LC-MS-UV analysis and the MarinLit database retrieval (http://pubs.rsc.org/marinlit, 15 June 2021) using the *m*/*z* values of 389.067 and 425.124 suggested the reasonable candidate molecules heraclemycin B [8] and bleomycin B [9]. Further analysis of the related molecular cluster indicated a series of putative new saliniquinone-related analogues through MarinLit database and SciFinder searches. Guided by LC-MS-UV, three undescribed saliniquinones, named saliniquinones G-I (**1**–**3**), and a new heraclemycin E (**4**) were obtained by repeated separation by column chromatography using silica gel, LH-20, and HPLC with an ODS column.

**Figure 3:**
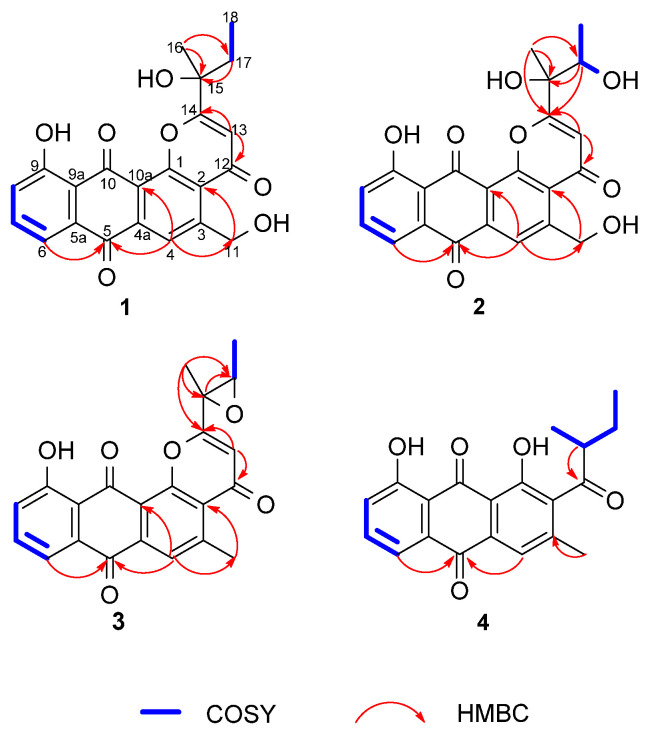
The key HMBC and COSY correlations in **1**–**4**.

Saliniquinone G (**1**) was obtained as yellow powder with a molecular formula of C_22_H_18_O_7_ deduced by HRESIMS, indicating fourteen degrees of unsaturation. The 1D NMR data of **1** (Table 1 and Table 2) are similar to those of saliniquinone F. [1] The difference was the replacement of methyl at C-5 in saliniquinone F [1] by a hydroxymethyl (C-11, *δ*_C_ 62.8, H_2_-11 *δ*_H_ 5.18) group, which was supported by the COSY correlation from OH-11 (*δ*_H_ 5.74) to H-11 (*δ*_H_ 5.18) and the HMBC correlation from H-11 to C-3 (*δ*_C_ 153.9), as well as the replacement of an allyl group on C-15 by an ethyl group (Table 1 and Table 2, Figure 3). The absolute configuration of C-15 was determined as 15*S* based on the CD data, which showed two negative Cotton effects at 267 nm and 372 nm (Figure S4), similar to those of saliniquinone F [1].

**Figure 4:**
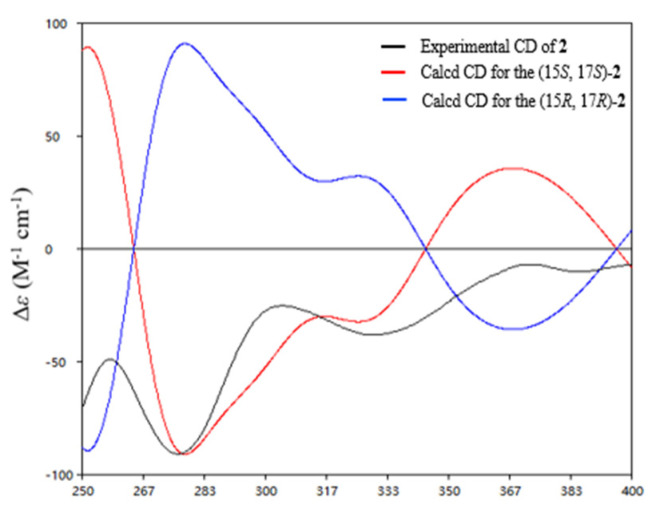
Experimental ECD spectra of compounds **2** and the calculated spectra for (15*S*, 17*S*)-**2**.

Saliniquinone H (**2**), obtained as red-yellow powder, has a molecular formula of C_22_H_18_O_8_, according to the (−)-HRESIMS *m*/*z* 409.0931 [M−H]^−^ (calcd. for C_22_H_17_O_8_, 409.0929). Examination of the NMR data (Table 1 and Table 2) showed considerable resemblance to those of **1**. The differences between **2** and **1** were the presence of an additional hydroxyl group at C-17 (*δ*_C_ 70.9) and the absence of one methylene on the side chain at C-15 (*δ*_C_ 76.5), which was supported by the downfield shift of C-17 (Table 2) and the COSY correlation from 17-OH (*δ*_H_ 4.67)/H-17(*δ*_H_ 4.20)/H_3_-18 (*δ*_H_ 1.20) (Table 1, Figure 3), as well as HMBC correlations from H-18 to C-15 (*δ*_C_ 76.5) and C-17, H-17 to C-14 (*δ*_C_ 174.9), C-15, and C-16 (*δ*_C_ 23.7), and H_3_-16 (*δ*_H_ 1.51) to C-14, C-15, and C-17. However, it was a challenge to determine the absolute configurations of C-15 and C-17 due to a free rotation of the C15–C17 single bond. Detailed analysis the ECD curve of **1** and saliniquinone C [1] allowed us to draw the conclusion that the negative Cotton effect around 263 nm and 372 nm indicated an *S* configuration. Accordingly, the hydroxy stereocenter at C-15 was an *S* configuration due to its negative Cotton effect around 263 nm and 372 nm. Hence, there are two relative configurations, named (15S*, 17S*)-**2a** and (15S*, 17R*)-**2b**, theoretically. The ^13^C NMR chemical shifts for the two possible isomers were calculated at the B3LYP/6-31+G(d)//B3LYP/6-311+G(d,p) levels and further checked by DP4+ probability [10,11]. The (15*S*, 17*S*)-**2a** isomer showed a striking predominance (100% probability) over the (15*S*, 17*R*)-**2b** isomer (Figure S6), which allowed us to assign the relative configuration of **2** as 15S*, 17S*. To determine the absolute configuration of C-15 and C-17 in **2**, the ECD calculations of the optimized conformation of (15*S*, 17*S*)-**2** obtained at the B3LYP/6-31+G(d) level were performed. The overall pattern of the experimental ECD spectrum was in reasonable agreement with the calculated one of (15*S*, 17*S*)-**2** (Figure 4), indicating the absolute configuration of C-15 and C-17 in **2** as 15*S*, 17*S*.

**Figure 5:**
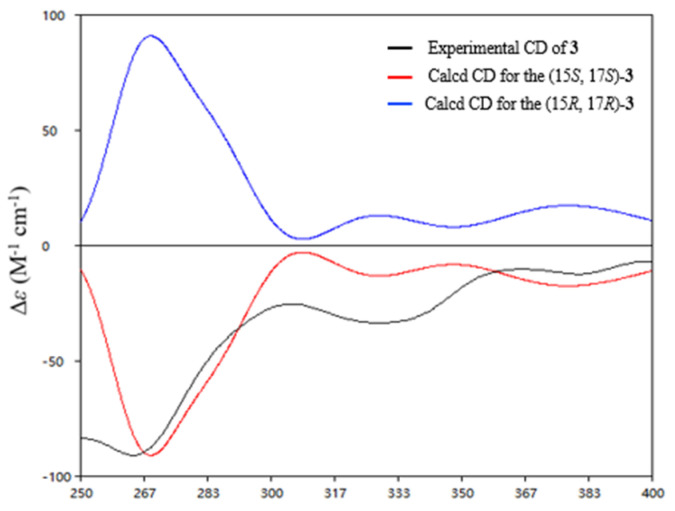
Experimental ECD spectra of compounds **3** and the calculated spectra for (15*S*, 17*S*)-**3**.

Saliniquinone I (**3**) was obtained as yellow powder with a molecular formula of C_22_H_16_O_6_ by HRESIMS. The 1D (Table 1 and Table 2) and 2D NMR (Figure 3) data indicated that **3** shares the same skeleton as **2**. Instead of the hydroxymethyl group in **2**, **3** has a methyl group (C-11, *δ*_C_ 24.1) at C-3, which was supported by HMBC correlation from H_3_-11 (*δ*_H_ 2.93) to C-3 (*δ*_C_ 156.4) (Figure 3), and possesses an epoxide ring between C-15 (Figure 3) and C-17, which is in agreement with the molecular formula as well as higher chemical shift values of C-17 (*δ*_C_ 62.2 in **3** vs. 70.9 in **2**) and C-15 (*δ*_C_ 59.9 in **3** vs. 76.5 in **2**). The relative configurations of C-15 and C-17 in **3** was evidenced by the NOESY correlations from H-17 (*δ*_H_ 3.48) to H_3_-16 (*δ*_H_ 1.85), which indicated 15S* and 17S* relative configurations of **3**. To determine the absolute configurations of C-15 and C-17, the optimized conformations of (15*S*, 17*S*)-**3** were obtained at the B3LYP/6-31+G(d) level and used for ECD calculations. The agreement of the experimental and calculated ECD curves (Figure 5) indicated the 15*S* and 17*S* absolute configurations of **3**.

**Figure 6:**
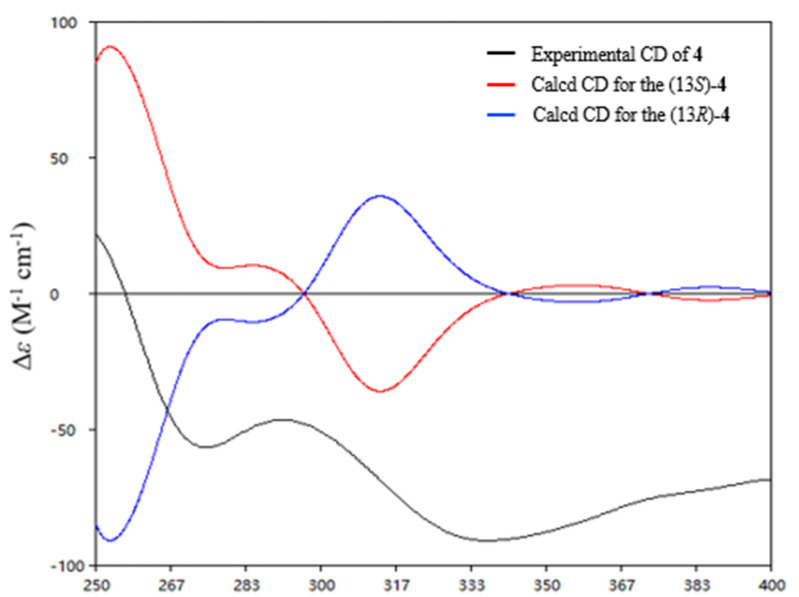
Experimental ECD spectrum of compound **4** and the calculated spectra for (13*S*)-**4**.

Heraclemycin E (**4**) was obtained as a brownish oil with a molecular formula of C_20_H_18_O_5_, as evidenced by HRESIMS. Comparison of the ^1^H and ^13^C NMR data of **4** with those of the reported heraclemycin C [4] revealed that they shared a similar anthraquinone skeleton. The difference between heraclemycin C and **4** is the substituent on C-2, being 2-methylhexanoyl in the former and 2-methylbutanoyl in the latter. This was confirmed by the COSY correlations from H-14 (*δ*_H_ 1.07)/H-13 (*δ*_H_ 3.00)/H-15 (*δ*_H_ 1.73, 1.34)/H-16 (*δ*_H_ 0.88) and HMBC correlations from H-13, H-14, and H-15 to C-12 (*δ*_C_ 208.9). The absolute configuration of C-13 was determined to be *S* in **4** by comparison of the calculated and experimental ECD spectra of 13*S*-**4** (Figure 6).

The new compounds (**1**–**4**) were evaluated for antibacterial activity against six bacterial strains, including methicillin-resistant coagulase-negative *staphylococci* (MRCNS), *B. subtilis*, *Proteus* sp., *B. cereus*, *Escherichia coli*, and *Mycobacterium phlei* [12]. Compounds **1** and **2** showed inhibitory effects against six strains, with MIC values ranging from 3.1 to 12.5 μM (Table 3). The structure activity relationship indicated the extra hydroxyl group at C-17 seems to play an important role for the inhibition activity (**1** vs. **2**). It was noted that the MIC values of **1** and **2** against MRCNS were 8-fold stronger than that of the positive control, ciprofloxacin (CPFX) [13].

## 3. Materials and Methods

### 3.1. General Experimental Procedures

The UV spectra were recorded on a Hitachi 5430 spectrophotometer (Hitachi Ltd., Tokyo, Japan). The ECD spectra and optical rotations were measured on a JASCO J-715 spectropolarimeter and a JASCOP-1020 digital (JASCO Corporation, Tokyo, Japan) polarimeter, respectively. IR spectra were obtained on a Bruker Tensor-27 (Bruker Corporation, Billerica, MA, USA). spectrophotometer in KBr discs. HRESIMS data were measured on a Thermo Scientific LTQ Orbitrap XL mass spectrometer (Thermo Fisher Scientific, Waltham, MA, USA). NMR spectra were collected on JEOLJN M-ECP 600 (JEOL Ltd., Tokyo, Japan and Agilent 500 MHz DD2 spectrometers (Agilent Technologies, Palo Alto, CA, USA), and tetramethylsilane was used as an internal standard. Sephadex LH-20 (Amersham Biosciences, NJ, USA) and silica gel (Qingdao Marine Chemical Factory, Qingdao, China) were used as stationary phases in column chromatography. An ODS column (YMC-Pack ODS-A, 10 × 250 mm, 5 μm, 3 mL/min, YMC Co., Ltd., Kyoto, Japan) was used for HPLC.

### 3.2. Actinomycete Material and Fermentation

*Nocardiopsis aegyptia* HDN19-252 (GenBank No. MN822699) was isolated from an animal sample collected from Antarctica (61°42′28″ S, 57°38′22″ W). The strain was aerobic and Gram-positive and produced beige to light-yellow aerial mycelium, brown substrate mycelium, and straight to flexuous hyphae but no specific spore chains [14]. It was deposited at the Key Laboratory of Marine Drugs, the Ministry of Education of China, School of Medicine and Pharmacy, Ocean University of China, Qingdao, People’s Republic of China.

*Nocardiopsis aegyptia* HDN19-252 was cultured in 1 L Erlenmeyer flasks containing 200 g of culture medium composed of 80 g of rice and 120 g of seawater, pH = 7.0 (in seawater collected from Huiquan Bay, Yellow Sea) at 28 °C for 25 days on stable fermentation. A total of 130 bottles of the culture medium were extracted with EtOAc (3 × 20 L) to generate a crude extract (10.2 g).

### 3.3. LC-MS/MS and Molecular Networking Analysis

LC-MS/MS analysis was performed using a UHPLC system (Ultimate 3000, Thermo Scientific) combined with a hybrid Quadrupole-Orbitrap mass spectrometer (QExactive, Thermo Scientific). As a mobile phase, 0.1% formic acid in H_2_O (A) and HPLC-grade MeCN (B) were used in negative-ionization conditions. The elution gradient conditions of LC-MS/MS were as follows, based on times (t): t = 0–1 min, hold at 10% B; t = 1–23 min, increased to 100% B linearly; t = 23–26 min, hold at 100% B; t = 26–30 min, returned to initial conditions and hold at 10% B to re-equilibrate the column. The elution velocity and injection volume were 0.25 mL/min and 3 μL, respectively. All MS/MS data were converted to mzXML format files by MSConvert software (Ver. 3.0.20169, MSConvert, ProteoWizard). Molecular networking was established by GNPS data analysis workflow and algorithms. The spectral network files were visualized through Cytoscape (Ver. 3.8.0, Cytoscape, NRNB.)

### 3.4. Isolation and Purification of Compounds

The crude extract was applied over a VLC column and eluted with mixtures of CH_2_Cl_2_-MeOH to give nine fractions (Fr.1–Fr.9). Fr.3–Fr.7 was combined as Fr.A, which was separated by HPLC using an ODS column to obtain ten subfractions (Fr.A.1–Fr.A.10). Fr.A.6 was purified by semi-preparative HPLC to obtain **2** (3 mg, *t*_R_ = 15 min). Fr.A.7 was purified by semi-preparative HPLC to afford **4** (2.5 mg, *t*_R_ = 13 min). Fr.A.8 was separated on the LH-20 column to obtain three subfractions (Fr.A.8.1–Fr.A.8.5). Fr.A.8.3 was purified by semi-preparative HPLC using a stepped gradient elution to obtain **1** (1.5 mg, *t*_R_ = 25 min). Fr.A.8.2 was purified by semi-preparative HPLC to afford **3** (2.1 mg, *t*_R_ = 27 min).

**Saliniquinone G** (**1**): yellow powder, [*α*]${}_{D}^{25}$ −12 (MeOH); UV (MeOH) *λ*_max_ 240 (1.6), 417 (0.3) nm; IR (KBr) *ν*_max_ 3414, 2926, 1679, 1211, 1139 cm^−1^; ECD (*c* 1.5mM, DMSO *λ*_max_ (Δ *ε*) 264 (−1.01), 372 (−0.22) nm; ^1^H and ^13^C NMR data, Table 1 and Table 2; HRESIMS *m*/*z* 393.0978 [M−H]^−^ (calcd for C_22_H_17_O_7_, 393.0980).

**Saliniquinone H** (**2**): red-yellow powder, [*α*]${}_{D}^{25}$ −83 (MeOH); UV (MeOH) *λ*_max_ (log *ε*) 240 (1.8), 419 (0.3) nm; IR (KBr) *ν*_max_ 3409, 2927, 1687, 1210, 1138 cm^−1^; ECD (*c* 1.5mM, DMSO *λ*_max_ (Δ *ε*) 264 (−8.63), 335 (−3.32) nm, 385 (−1.02) nm; ^1^H and ^13^C NMR data, Table 1 and Table 2; HRESIMS *m*/*z* 409.0931 [M−H]^−^ (calcd for C_22_H_17_O_8_, 409.0929).

**Saliniquinone I** (**3**): yellow powder, [*α*]${}_{D}^{25}$ −83 (MeOH); UV (MeOH) *λ*_max_ 241 (1.5), 417 (0.3) nm; IR (KBr) *ν*_max_ 3437, 2925, 1679, 1215, 1140 cm^−1^; ECD (*c* 1.5mM, DMSO *λ*_max_ (Δ *ε*) 264 (−4.00), 335 (−1.37) nm, 385 (−0.53) nm; ^1^H and ^13^C NMR data, Table 1 and Table 2; HRESIMS *m*/*z* 375.0881 [M−H]^−^ (calcd for C_22_H_15_O_6_, 375.0874).

**Heraclemycin E** (**4**): brownish oil, [*α*]${}_{D}^{25}$ −12 (MeOH); UV (MeOH) *λ*_max_ 225 (0.5), 380 (0.3) nm; IR (KBr) *ν*_max_ 3435, 2929, 1696, 1210, 1156 cm^−1^; ECD (*c* 1.5mM, DMSO *λ*_max_ (Δ *ε*) 264 (−0.91), 335 (−0.86) nm; ^1^H and ^13^C NMR data, Table 1 and Table 2; HRESIMS *m*/*z* 337.1075 [M−H]^−^ (calcd for C_20_H_17_O_5_, 337.1081).

### 3.5. Computation Section

Conformational searches were run, employing Spartan’14, [15] based on the MMFF (Merck Molecular Force Field). All conformers were further optimized with DFT calculations at the B3LYP/6-31+G(d) level by using the Gaussian 09 program [16]. TDDFT calculations were performed on the five lowest-energy conformations for **2**, the lowest-energy conformation for **3**, and the six lowest-energy conformations for **4** (>5% population). ECD spectra were obtained on the program SpecDis 1.71 software [17] by using a Gaussian band shape with a 0.25 eV width for **2**, a 0.3 eV width for **3,** and a 0.25 eV width for **4** from dipole-length rotational strengths. The calculated spectra were shifted by −25 nm for **2**, 32 nm for **3,** and 0 nm for **4** to facilitate comparison to the experimental data.

### 3.6. Assay of Antimicrobial Activity

Antibacterial activity of **1**–**4** was evaluated against MRCNS, *B. subtilis*, *Proteus* sp., *B. cereus*, *Escherichia coli*, *Mycobacterium phlei* by a conventional broth dilution assay. Six strains were cultured in 100 mL Erlenmeyer flasks at 28 °C for 24 h. Then, the culture medium was diluted to a concentration of 10^6^ cfu/mL and added into 96-well plates. Ciprofloxacin was used as a positive control. The detailed methodologies for biological testing have been described in previous reports [14].

## 4. Conclusions

In summary, four new anthraquinone derivatives were isolated from *Nocardiopsis aegyptia* HDN19-252 under the guidance of GNPS. Compared with saliniquinone I (**3**), saliniquinone G (**1**) and saliniquinone H (**2**) exhibited significant antibacterial activity against six tested bacterial strains, suggesting that a free hydroxyl group is an important part of antibacterial activity. Compounds **1**–**3** represent a rare class of saliniquinones with *S* configuration at C-15, indicating a stereospecific ketoreductase in strain *N. aegyptia* HDN19-252. Additionally, this is also the first report of saliniquinones from *Nocardia* sp. Notably, **1** and **2** specifically inhibited the growth of a drug-resistant MRCNS strain with an MIC of 6.2 μM, which was even stronger than the positive control, CPFX (50 μM). Our results highlight the potential for screening and developing therapeutic molecules from actinomycete-derived saliniquinones.

## Figures and Tables

**Table 1:** ^1^H NMR (600 MHz) spectroscopic data of **1**–**4** in DMSO-*d*_6_ (*δ* in ppm, *J* in Hz).

No.	1	2	3	4
4	8.55, s	8.55, s	8.01, s	7.60, s
6	7.73, d (7.5)	7.74, d (7.0)	7.73, d (6.3)	7.70, d (7.5)
7	7.81, t (8.0)	7.81, t (7.2)	7.81, t (8)	7.79, t (7.4)
8	7.43, d (8.8)	7.44, d (7.0)	7.44, d (8.4)	7.38, d (8.4)
11	5.18, d (6.3)	5.19, d (4.0)	2.93, s	2.28, s
13	6.49, s	6.54, s	6.22, s	3.00, m
14	-	-	-	1.07, d (7.2)
15	-	-	-	1.73, m, 1.34, m
16	1.61, s	1.51, s	1.85, s	0.88, t (7.5)
17	1.84, m 2.07, m	4.20, m	3.48, q (5.0)	-
18 11-OH 15-OH 17-OH	0.83, t (7.5) 5.74, t 5.59, s -	1.20, d (6.0) 5.73, t 5.42, s 4.67, d	1.22, d (5.2) - - -	- - - -

**Table 2:** ^13^C NMR (150 MHz) spectroscopic data of **1**–**4** in DMSO-*d*_6_ (*δ* in ppm).

No.	1	2	3	4
1	174.7, C	174.9 C	176.0, C	158.7, C
2	124.5, C	124.6, C	126.3, C	145.5, C
3	153.9, C	153.9, C	156.4, C	136.0, C
4 4a	119.3, CH 120.5, C	119.4, CH 120.5, C	125.6, CH 120.5, C	121.8, CH 114.9, C
5 5a	182.5, C 132.9, C	182.3, C 132.9, C	182.1, C 132.8, C	181.7, C 133.9, C
6	119.3, CH	119.3, CH	119.3, CH	120.0, CH
7	137.3, CH	137.3, CH	137.5, CH	138.1, CH
8	125.3, CH	125.3, CH	125.4, CH	125.1, CH
9 9a	161.9, C 117.5, C	163.3, C 117.4, C	161.8, C 117.4, C	161.9, C 116.6, C
10 10a	187.8, C 136.8, C	187.7, C 136.7, C	187.8, C 137.3, C	192.3, C 153.2, C
11	62.8, CH_2_	62.9, CH_2_	24.1, CH_3_	20.3, CH_3_
12	179.0, C	179.1, C	178.5, C	208.9, C
13	109.4, CH	110.2, CH	111.2, CH	48.2, CH
14	174.7, C	174.9, C	165.7, C	15.0, CH_3_
15	73.3, C	76.5, C	59.9, C	24.9, CH_2_
16	27.4, CH_3_	23.7, CH_3_	20.8, CH_3_	12.0, CH_3_
17	33.6, CH_2_	70.9, CH	62.2, CH	-
18	8.5, CH_3_	17.4, CH_3_	13.7, CH_3_	-

**Table 3:** Inhibition effects of **1**–**4** against six pathogenic bacteria.

Compd.	MIC (μM)					
	MRCNS	*B. subtilis*	*P. species*	*B. cereus*	*E. coli*	*M. Phlei*
1	6.2	6.2	12.5	6.2	6.2	6.2
2	6.2	6.2	6.2	6.2	6.2	3.1
3	>50	>50	>50	>50	>50	>50
4	>50	>50	>50	>50	>50	>50
CPFX	50	0.01	0.2	3.1	3.1	1.5

## Data Availability

The data presented in this study are available in this article and supplementary material.

## Supplementary Materials

The following are available online at https://www.mdpi.com/article/10.3390/md19100575/s1, Figure S1: the picture of Antarctica animal, Figure S2: *Nocardiopsis aegyptia* HDN19-252; Figure S3: HPLC analysis of the crude extract of HDN19-252; Figure S4: the experimental curves of **1** and saliniquinones F; Figure S5: correlation plots of experimental ^13^C NMR chemical shifts versus the corresponding calculated data for **2a** and **2b**; Figure S6: sDP4+, uDP4+ and DP4+ probabilities (%) for compound **2a** and **2b**; Figure S7-S38: 1D and 2D NMR spectra, HRESIMS spectra, IR spectra of compounds **1**–**4**; Table S1: deviations between the calculated and experimental ^13^C NMR chemical shifts for stereoisomers **2a** and **2b**.
