# Supplementary material for: Saliniquinone Derivatives, Saliniquinones G−I and Heraclemycin E, from the Marine Animal-Derived Nocardiopsis aegyptia HDN19-252
Source: Mar Drugs. 2021 Oct 14;19(10):575. doi: 10.3390/md19100575 (PMC8537165; doi:10.3390/md19100575)
Supplement: Supplementary file 1 [file marinedrugs-19-00575-s001.zip › marinedrugs-1410887-supplementary.pdf]

Supporting Information for

Saliniquinone Derivatives, Saliniquinones G–I and Heraclemycin E, from the Marine Animal-derived *Nocardiopsis aegyptia* HDN19-252

Luning Zhou [1], Xuedong Chen [1], Chunxiao Sun [1], Yimin Chang [1], Xiaofei Huang [1], Tianjiao Zhu [1], Guojian Zhang [1,2], Qian Che [1,*], and Dehai Li [1,2,*]

[1] School of Medicine and Pharmacy, Ocean University of China, Qingdao 266003, People's Republic of China; 18895692529@163.com (L.Z.); chenxuedong1206@163.com (X.C.); sunchunxiao93@163.com (C.S.); yiminchang@163.com (Y.C.); hxf17853102898@163.com (X.H.); zhutj@ouc.edu.cn (T.Z.); cheqian064@ouc.edu.cn (Q.C.); dehaili@ouc.edu.cn (D.L.)

[2] Laboratory for Marine Drugs and Bioproducts of Qingdao National Laboratory for Marine Science and Technology, Qingdao, 266237, People's Republic of China; zhangguojian@ouc.edu.cn (G.Z.); dehaili@ouc.edu.cn (D.L.)

* Correspondence: dehaili@ouc.edu.cn (D.L.); Tel.: 0086-532-82031619; cheqian064@ouc.edu.cn (Q. C.).

Table of Contents

Figure S1. The picture of Antarctica animal 4

Figure S2. *Nocardiopsis aegyptia* HDN19-252. 4

Figure S3. HPLC analysis of the crude extract of HDN19-252. 4

The 16S rRNA sequences data of *Nocardiopsis aegyptia* HDN19-252. 4

Figure S4. The experimental curves of **1** and saliniquinones F. 5

Figure S5. Correlation plots of experimental ^{13}C NMR chemical shifts versus the corresponding calculated data for **2a** and **2b**. 6

Figure S6. sDP4+, uDP4+ and DP4+ probabilities (%) for compound **2a** and **2b**. .. 6

Table S1. Deviations between the calculated and experimental ^{13}C NMR chemical shifts for stereoisomers **2a** and **2b**. 6

Figure S7. ^{1}H NMR (500 MHz, DMSO) spectrum of saliniquinone G (**1**). 7

Figure S8. ^{13}C NMR (150 MHz, DMSO) spectrum of saliniquinone G (**1**). 7

Figure S9. HSQC spectrum of saliniquinone G (**1**). 8

Figure S10. ^{1}H-^{13}C HMBC spectrum of saliniquinone G (**1**). 8

Figure S11. ^{1}H-^{1}H COSY spectrum of saliniquinone G (**1**). 9

Figure S12. NOESY spectrum of saliniquinone G (**1**). 9

Figure S13. HRESIMS spectrum of saliniquinone G (**1**). 10

Figure S14. IR spectrum of saliniquinone G (**1**). 10

Figure S15. ^{1}H NMR (400 MHz, DMSO) spectrum of saliniquinone H (**2**). 11

Figure S16. ^{13}C NMR (150 MHz, DMSO) spectrum of saliniquinone H (**2**). 11

Figure S17. HSQC spectrum of saliniquinone H (**2**). 12

Figure S18. ^{1}H-^{13}C HMBC spectrum of saliniquinone H (**2**). 12

Figure S19. ^{1}H-^{1}H COSY HMBC spectrum of saliniquinone H (**2**). 13

Figure S20. NOESY spectrum of saliniquinone H (**2**). 13

Figure S21. HRESIMS spectrum of saliniquinone H (**2**). 14

Figure S22. IR spectrum of saliniquinone H (**2**). 14

Figure S23. ^{1}H NMR (400 MHz, DMSO) spectrum of saliniquinone I (**3**). 15

Figure S24. ^{13}C NMR (150 MHz, DMSO) spectrum of saliniquinone I (**3**). 15

Figure S25. HSQC spectrum of saliniquinone I (**3**). 16

Figure S26. ^{1}H-^{13}C HMBC spectrum of saliniquinone I (**3**). 16

Figure S27. ^{1}H-^{1}H COSY spectrum of saliniquinone I (**3**). 17

Figure S28. NOESY spectrum of saliniquinone I (**3**). 17
Figure S29. HRESIMR spectrum of saliniquinone I (**3**). 18
Figure S30. IR spectrum of saliniquinone I (**3**). 18
Figure S31. ^{1}H NMR (600 MHz, DMSO) spectrum of heraclemycin E (**4**). 19
Figure S32. ^{13}C NMR (150 MHz, DMSO) spectrum of heraclemycin E (**4**). 19
Figure S33. HSQC spectrum of heraclemycin E (**4**). 20
Figure S34. ^{1}H-^{13}C HMBC spectrum of heraclemycin E (**4**). 20
Figure S35. ^{1}H-^{1}H COSY spectrum of heraclemycin E (**4**). 21
Figure S36. NOESY spectrum of heraclemycin E (**4**). 21
Figure S37. HRESIMS spectrum of heraclemycin E (**4**). 22
Figure S38. IR spectrum of heraclemycin E (**4**). 22
Figure S39. Cartesian coordinates of the low-energy reoptimized conformers of **2a** calculated at B3LYP/6-31+G(d) level of theory. 23
Figure S40. Cartesian coordinates of the low-energy reoptimized conformers of **2b** calculated at B3LYP/6-31+G(d) level of theory. 29

Figure S1. The picture of Antarctica animal

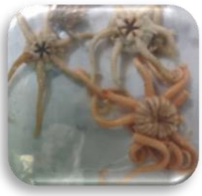

Figure S2. *Nocardiopsis aegyptia* HDN19-252.

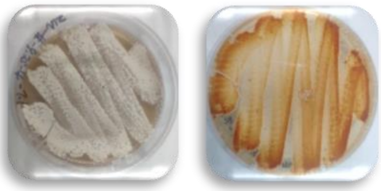

Figure S3. HPLC analysis of the crude extract of HDN19-252.

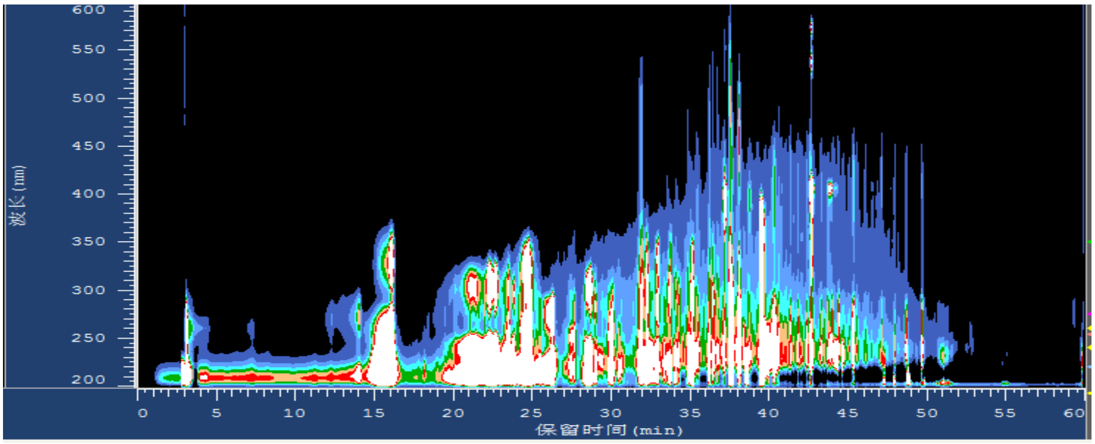

The 16S rRNA sequences data of *Nocardiopsis aegyptia* HDN19-252.

GGAAGTGCGGCGTGCTACACATGCAGTCGAGCGGTAAGGCCCTTCGGGGTACACGAG
CGGCGAACGGGTGAGTAACACGTGAGCAACCTGCCCCCGACTCCGGGATAAGCGGTG
GAAACGCCGTCTAATACCGGATACGACCGGCCACCTCATGGTAGCCGGTGGAAAGTTT
TTCGGTTGGGGATGGGCTCGCGGCCTATCAGCTTGTTGGTGGGGTAAAGGCCTACCAA
GGCGATTACGGGTAGCCGGCCTGAGAGGGCGACCGGCCACACTGGGACTGAGACACG
GCCCAGACTCCTGCGGGAGGCAGCAGTGGGGAATATTGCGCAATGGGCGAAAGCCTG
ACGCAGCGACGCCGCGTGGGGGATGACGGCCTTCGGGTTGTAAACCTCTTTTACCACC
AACGCAGGCTCCCAGTTCTCTGGGGGTTGACGGTAGGTGGGGAATAAGGACCGGCTA
ACTACGTGCCAGCAGCCGCGGTAATACGTAGGGTCCGAGCGTTGTCCGGAATTATTGG
GCGTAAAGAGCTCGTAGGCGGCGTGTCGCGTCTGCTGTGAAAGACCGGGGCTTAACTC
CGGTTCTGCAGTGGATACGGGCATGCTAGAGGTAGGTAGGGGAGACTGGAATTCCTGG
TGTAGCGGTGAAATGCGCAGATATCAGGAGGAACACCGGTGGCGAAGGCGGGTCTCT
GGGCCTTACCTGACGCTGAGGAGCGAAAGCATGGGGAGCGAACAGGATTAGATACCC
TGGTAGTCCATGCCGTAAACGTTGGGCGCTAGGTGTGGGGACTTTCCACGGTTTCCGC

GCCGTAGCTAACGCATTAAGCGCCCCGCCTGGGGAGTACGGCCGCAAGGCTAAAACTC
AAAGGAATTGACGGGGGCCCGCACAAGCGGCGGAGCATGTTGCTTAATTCGACGCAA
CGCGAAGAACCTTACCAAGGTTTGACATCACCCGTGGACCTGCAGAGATGTGGGGTCA
TTTAGTTGGTGGGTGACAGGTGGTGCATGGCTGTCGTCAGCTCGTGTCGTGAGATGTT
GGGTTAAGTCCCGCAACGAGCGCAACCCTTATTCCATGTTGCCAGCACGTAGTGGTGG
GGACTCATGGGAGACTGCCGGGGTCAACTCGGAGGAAGGTGGGGACGACGTCAAGTC
ATCATGCCCCTTATGTCTTGGGCTGCAAACATGCTACAATGGCCGGTACAATGGGCGTG
CGATACCGTAAGGTGGAGCGAATCCCTAAAAGCCGGTCTCAGTTCGGATTGGGGTCTG
CAACTCGACCCCATGAAGGTGGAGTCGCTAGTAATCGCGGATCAGCAACGCCGCGGTG
AATACGTTCCCGGGCCTTGTACACACCGCCCGTCACGTCATGAAAGTCGGCAACACCC
GAAACTTGTGGCCTAACCCTTCGGGGAGGGAATGAGGAAGTTGTCCGACTTT

Figure S4. The experimental curves of **1** and saliniquinones F.

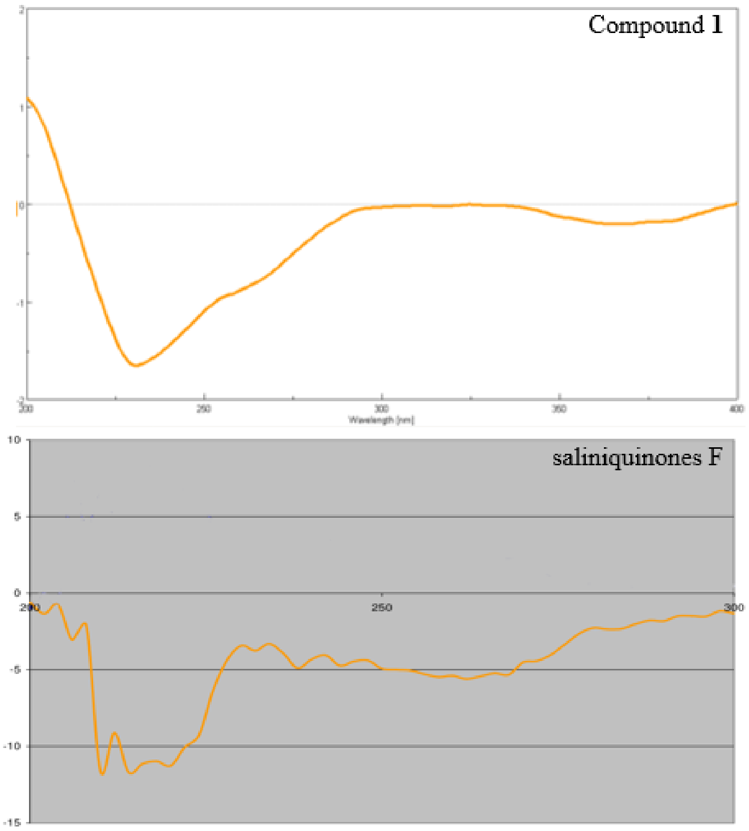

Figure S5. Correlation plots of experimental ^{13}C NMR chemical shifts versus the corresponding calculated data for **2a** and **2b**.

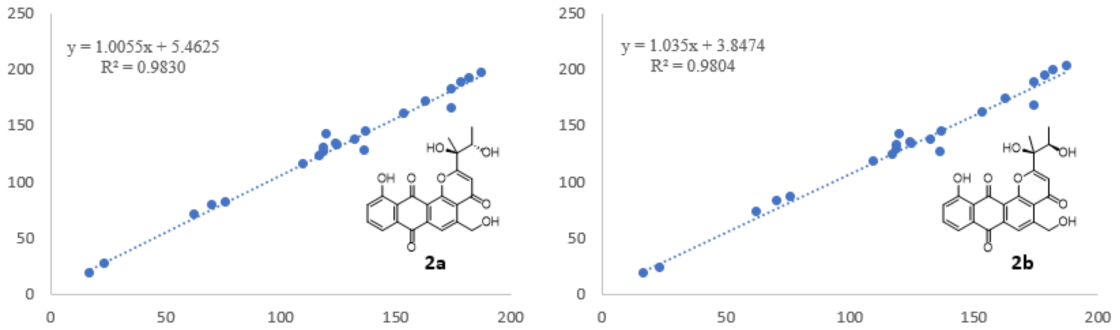

Figure S6. sDP4+, uDP4+ and DP4+ probabilities (%) for compound **2a** and **2b**.

Functional	Solvent?	Basis Set	Type of Data		
B3LYP	PCM	6-311G(d,p)	Shielding Tensors		
	Isomer 1	Isomer 2	Isomer 3	Isomer 4	Isomer 5	Isomer 6
sDP4+ (H data)	18.12%	81.88%	-	-	-	-
sDP4+ (C data)	100.00%	0.00%	-	-	-	-
sDP4+ (all data)	100.00%	0.00%	-	-	-	-
uDP4+ (H data)	99.96%	0.04%	-	-	-	-
uDP4+ (C data)	100.00%	0.00%	-	-	-	-
uDP4+ (all data)	100.00%	0.00%	-	-	-	-
DP4+ (H data)	99.80%	0.20%	-	-	-	-
DP4+ (C data)	100.00%	0.00%	-	-	-	-
DP4+ (all data)	100.00%	0.00%	-	-	-	-

Table S1. Deviations between the calculated and experimental ^{13}C NMR chemical shifts for stereoisomers **2a** and **2b**.

2a					2b				
Calcd.	Exp.	Scal.	Δδ	∣ Δδ ∣	Calcd.	Exp.	Scal.	Δδ	∣ Δδ ∣
171.6	163.3	165.2	-1.9	1.9	173.7	163.3	164.2	-0.9	0.9
132.6	125.3	126.4	-1.1	1.1	133.6	125.3	125.4	-0.1	0.1
144.1	137.3	137.9	-0.6	0.6	144.9	137.3	136.3	1.0	1.0
125.9	119.3	119.8	-0.5	0.5	128.3	119.3	120.2	-0.9	0.9
137.5	132.9	131.3	1.6	1.6	136.8	132.9	128.4	4.5	4.5
122.7	117.4	116.6	0.8	0.8	124.0	117.4	116.1	1.3	1.3
192.2	182.3	185.7	-3.4	3.4	198.7	182.3	188.3	-6.0	6.0
142.5	120.5	136.3	-15.8	15.8	141.7	120.5	133.2	-12.7	12.7
127.3	136.7	121.1	15.6	15.6	126.6	136.7	118.6	18.1	18.1
197.1	187.7	190.6	-2.9	2.9	202.8	187.7	192.2	-4.5	4.5
129.9	119.4	123.8	-4.4	4.4	132.0	119.4	123.8	-4.4	4.4
160.5	153.9	154.2	-0.3	0.3	161.7	153.9	152.5	1.4	1.4
133.5	124.6	127.3	-2.7	2.7	134.4	124.6	126.2	-1.6	1.6
164.9	174.9	158.6	16.3	16.3	167.6	174.9	158.2	16.7	16.7
188.4	179.1	181.9	-2.8	2.8	194.1	179.1	183.8	-4.7	4.7
115.9	110.2	109.8	0.4	0.4	117.7	110.2	110.0	0.2	0.2
182.3	174.9	175.9	-1.0	1.0	188.2	174.9	178.1	-3.2	3.2
81.9	76.5	76.0	0.5	0.5	86.5	76.5	79.9	-3.4	3.4
70.7	62.9	64.9	-2.0	2.0	72.6	62.9	66.4	-3.5	3.5

78.6	70.9	72.8	-1.9	1.9	82.4	70.9	75.9	-5.0	5.0
27.0	23.7	21.4	2.3	2.3	23.6	23.7	19.1	4.6	4.6
19.2	17.4	13.7	3.7	3.7	18.7	17.4	14.4	3.0	3.0
			MAE	3.7				MAE	4.6
			RMSE	6.2				RMSE	6.7
			R^2	0.9830				R^2	0.9804

Figure S7. ^{1}H NMR (500 MHz, DMSO) spectrum of saliniquinone G (**1**).

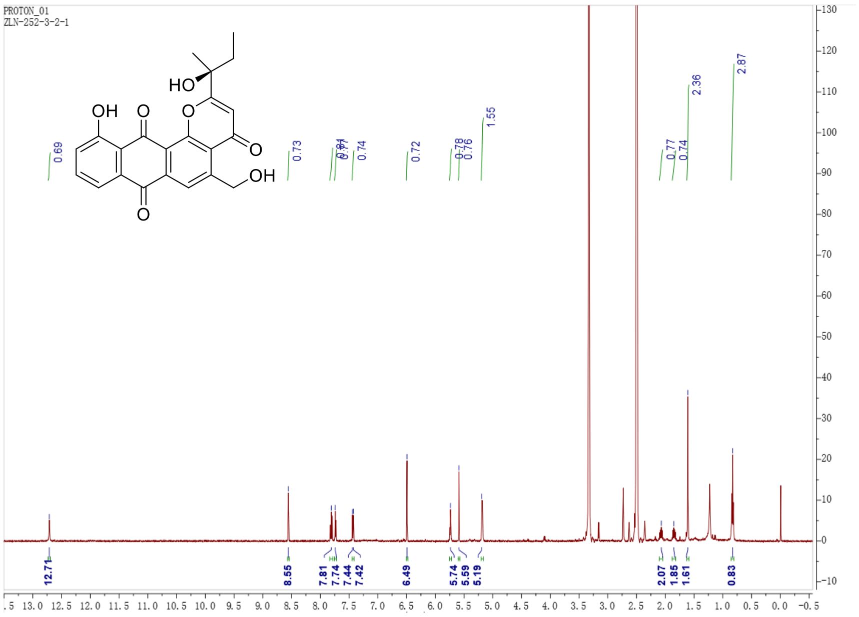

Figure S8. ^{13}C NMR (150 MHz, DMSO) spectrum of saliniquinone G (**1**).

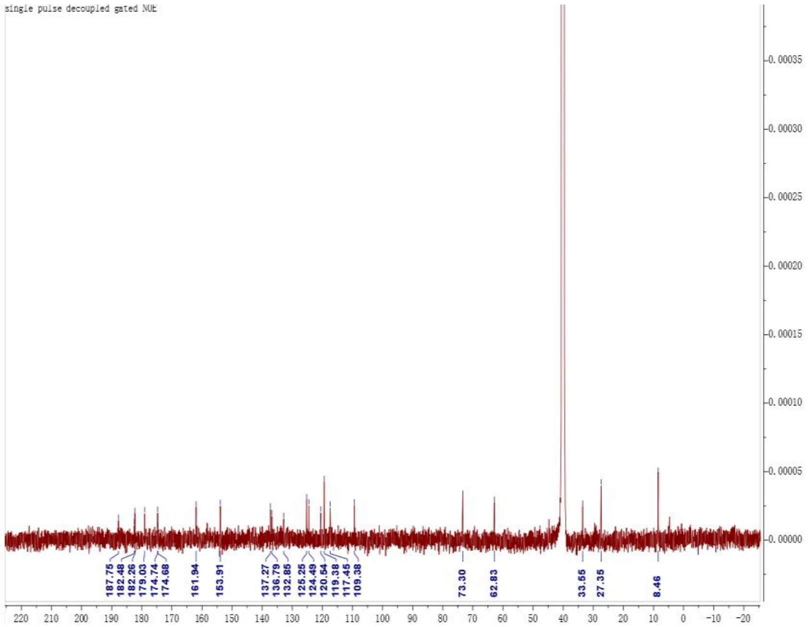

Figure S9. HSQC spectrum of saliniquinone G (**1**).

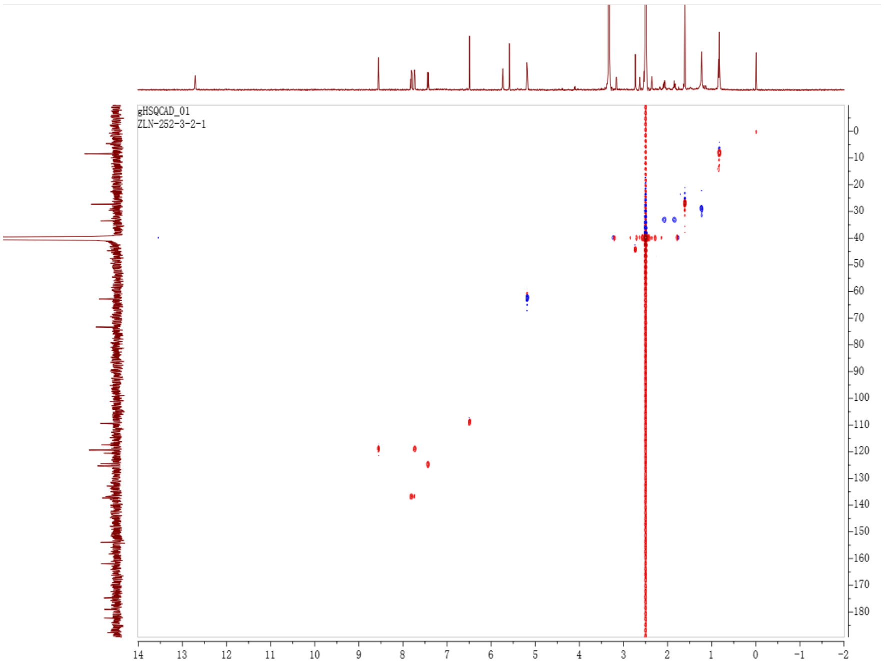

Figure S10. ^{1}H-^{13}C HMBC spectrum of saliniquinone G (**1**).

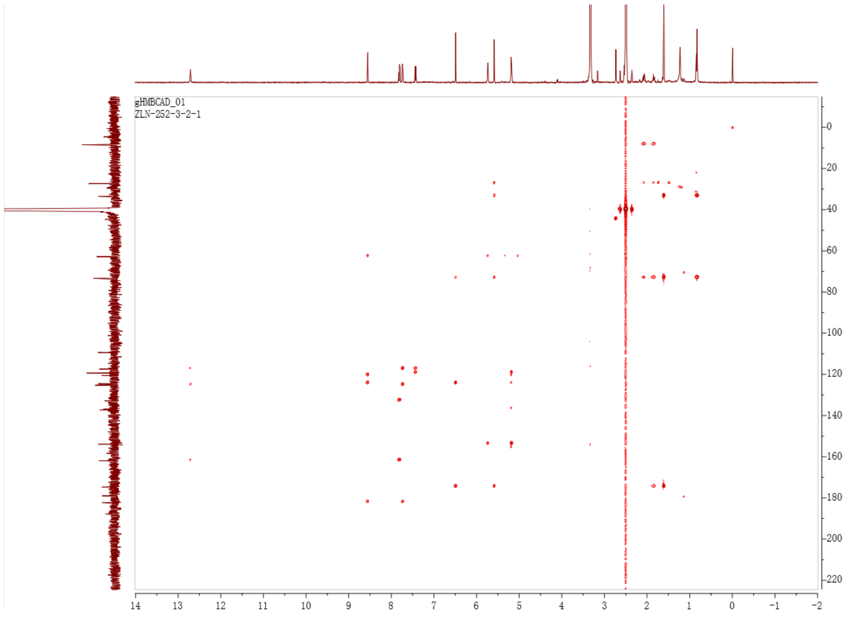

Figure S11. ^{1}H-^{1}H COSY spectrum of saliniquinone G (**1**).

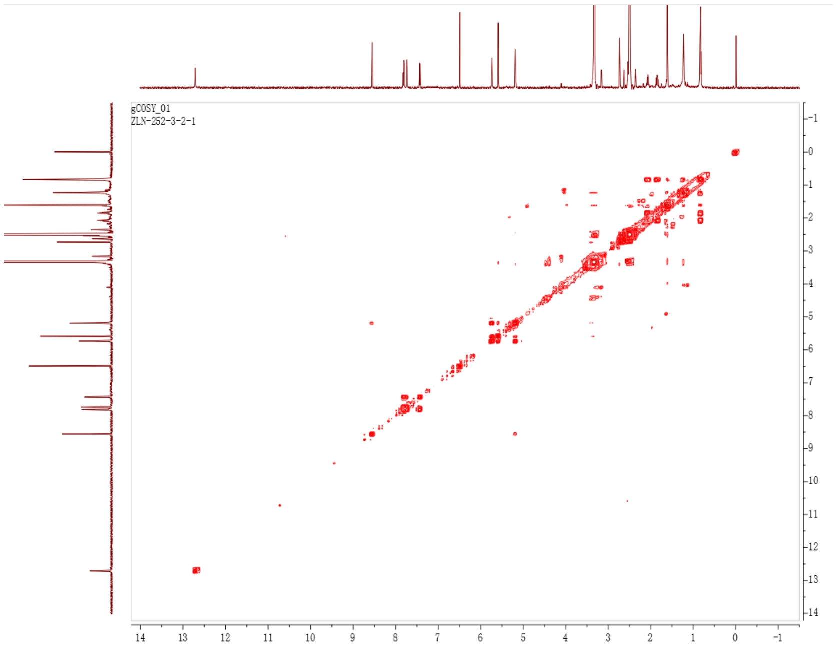

Figure S12. NOESY spectrum of saliniquinone G (**1**).

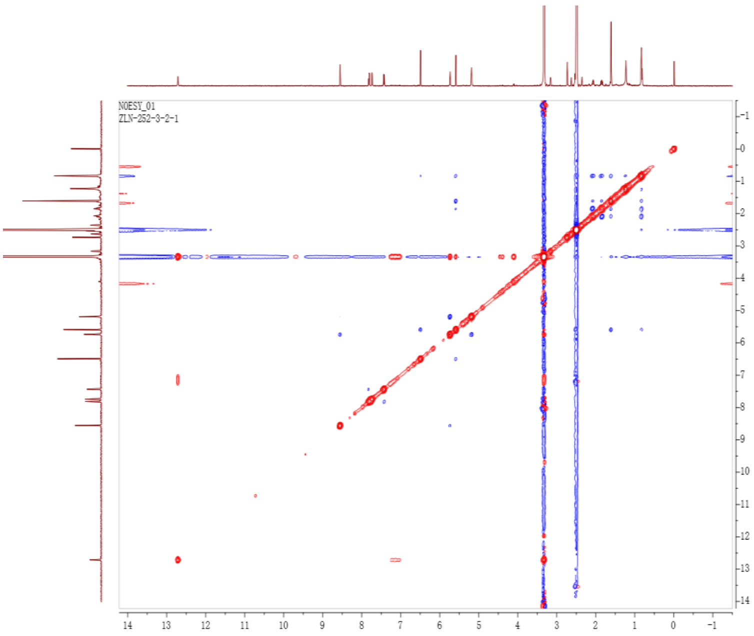

Figure S13. HRESIMS spectrum of saliniquinone G (**1**).

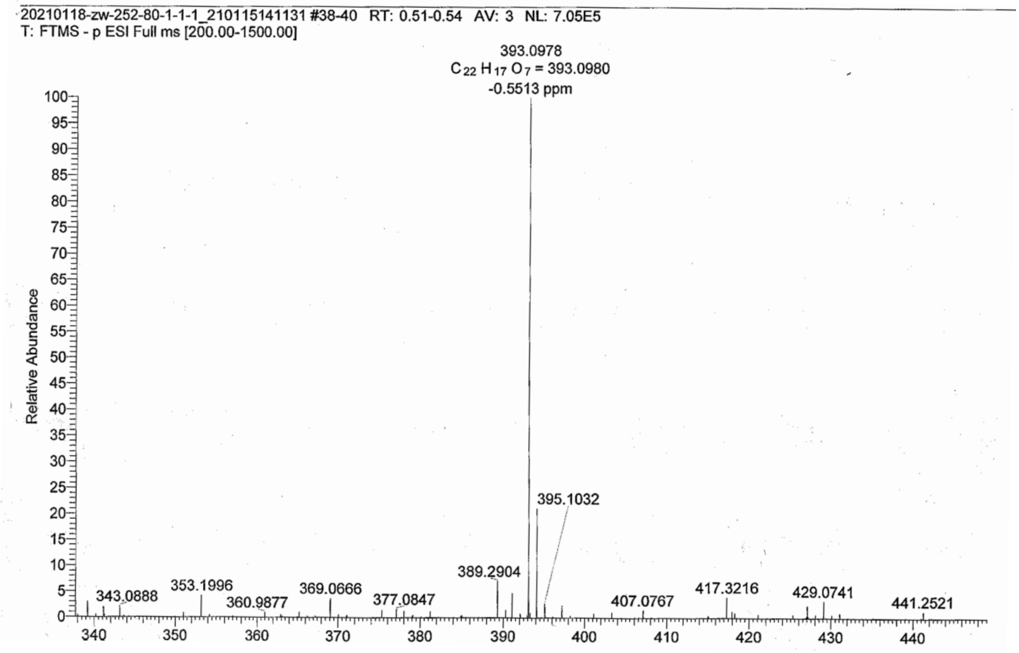

Figure S14. IR spectrum of saliniquinone G (**1**).

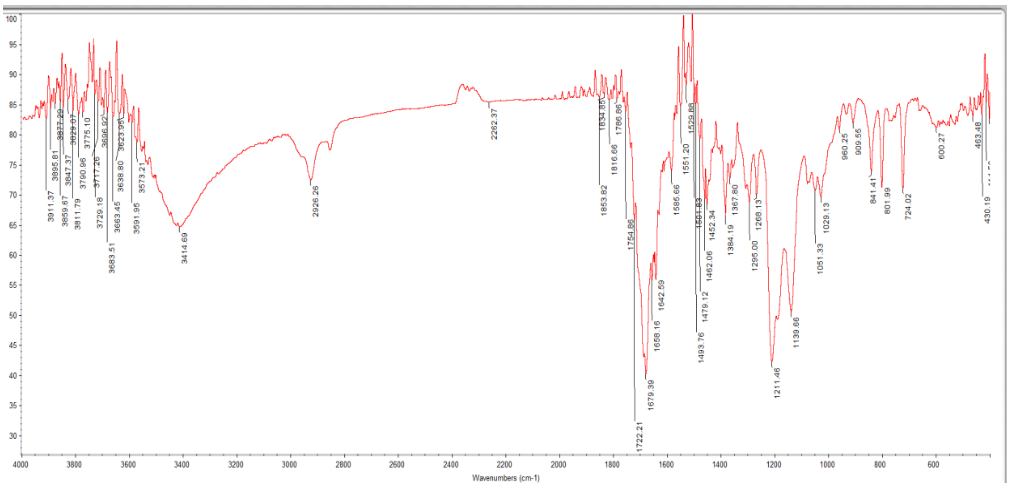

Figure S15. ^{1}H NMR (400 MHz, DMSO) spectrum of saliniquinone H (**2**).

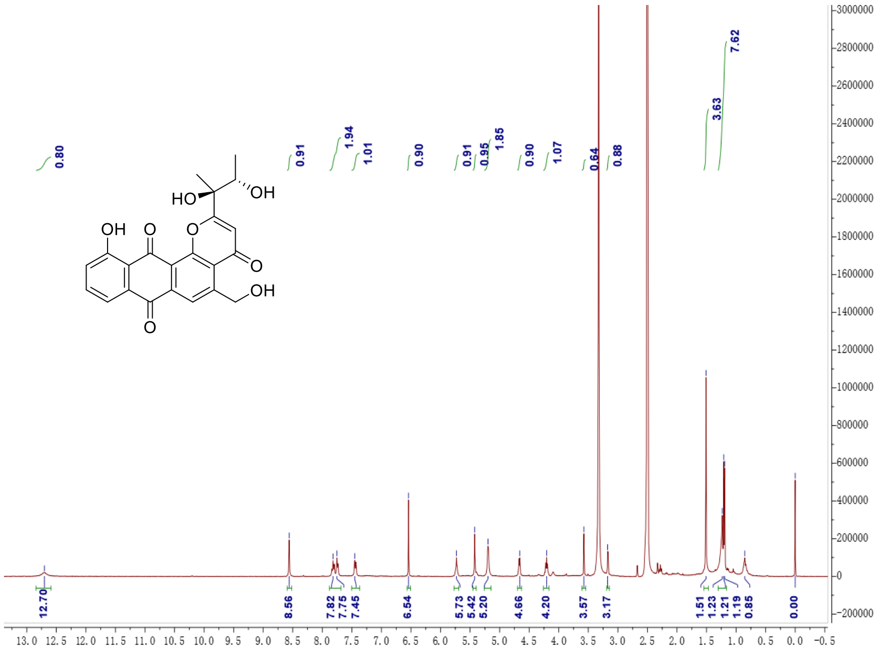

Figure S16. ^{13}C NMR (150 MHz, DMSO) spectrum of saliniquinone H (**2**).

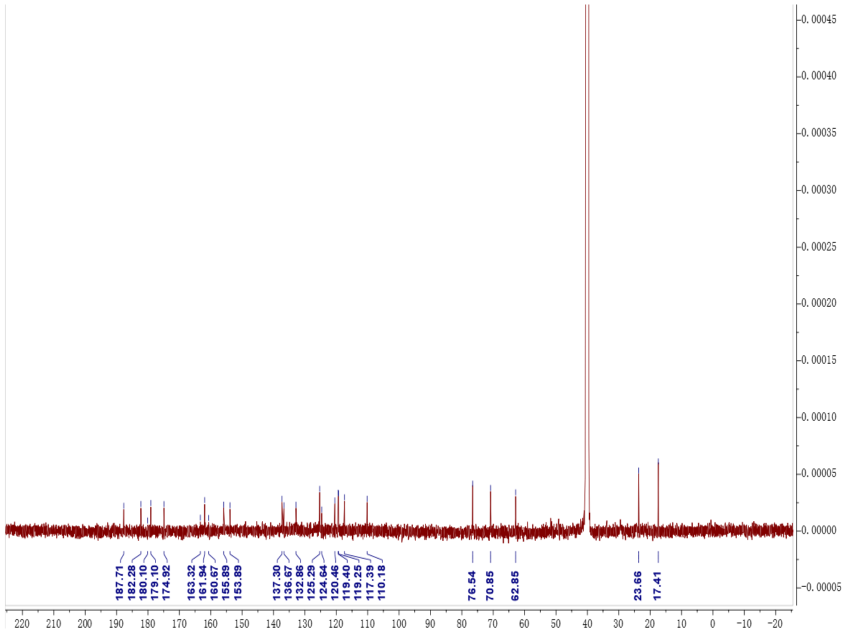

Figure S17. HSQC spectrum of saliniquinone H (**2**).

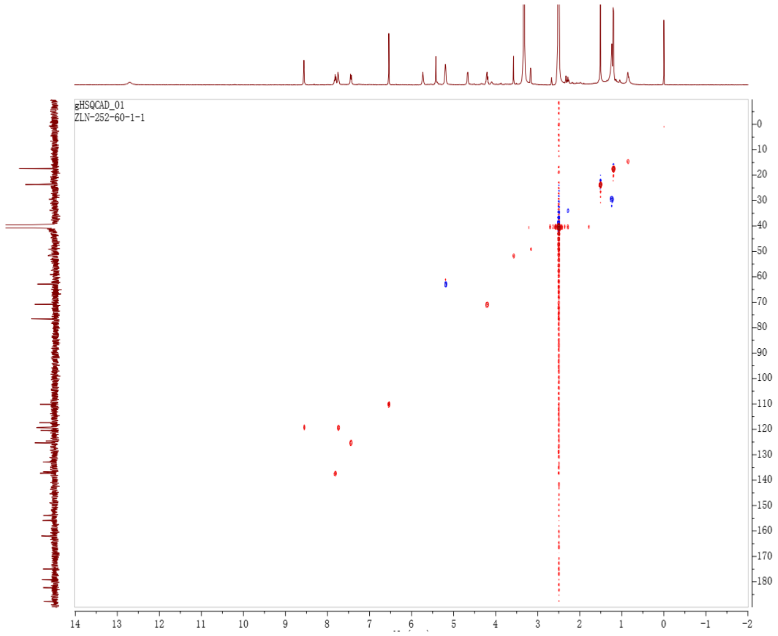

Figure S18. ^{1}H-^{13}C HMBC spectrum of saliniquinone H (**2**).

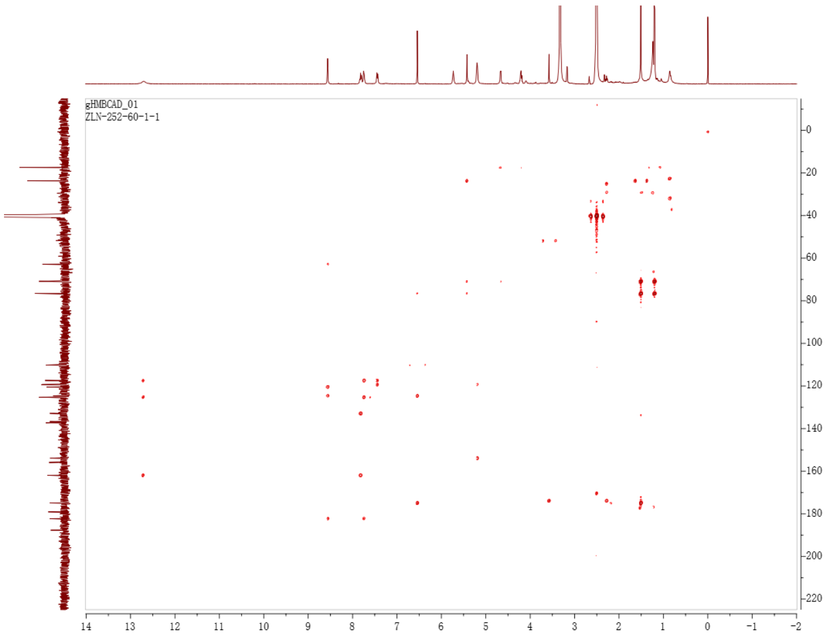

Figure S19. ^{1}H-^{1}H COSY HMBC spectrum of saliniquinone H (**2**).

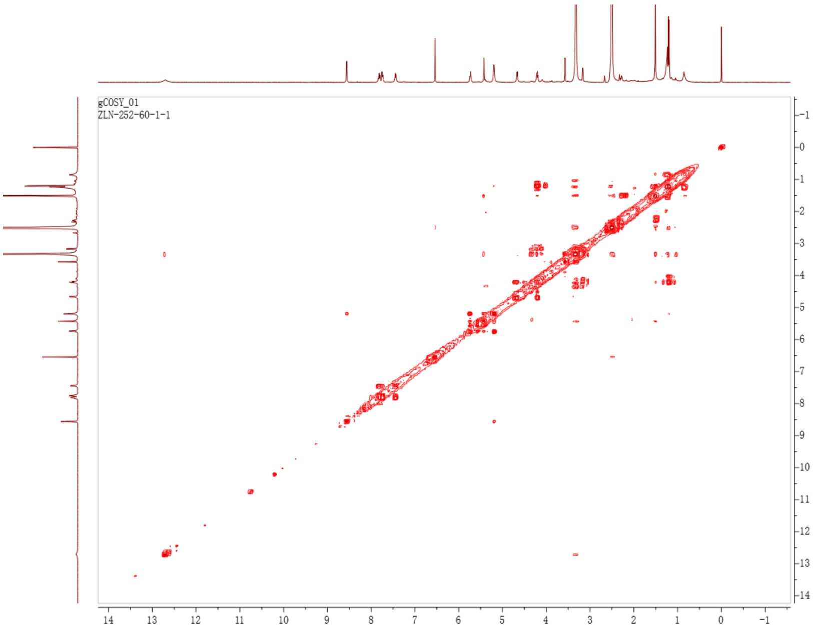

Figure S20. NOESY spectrum of saliniquinone H (**2**).

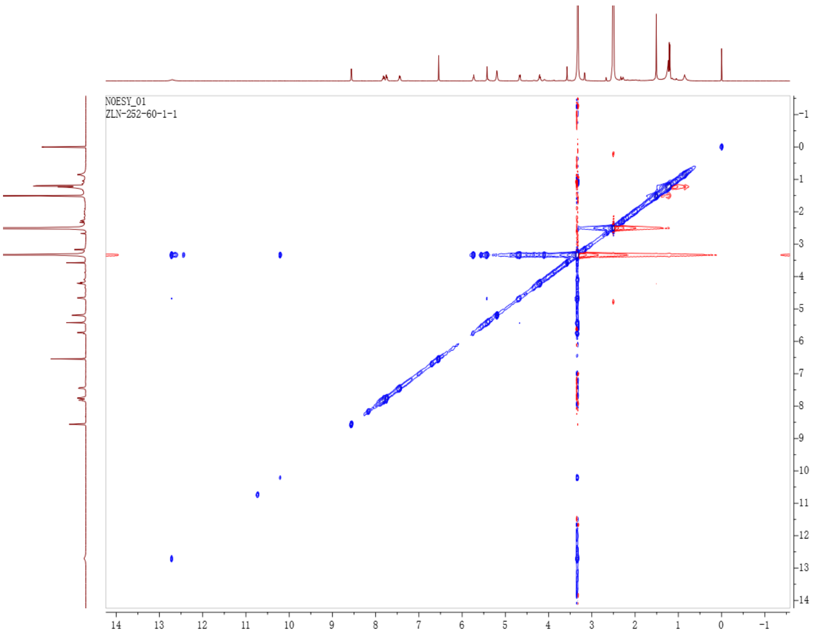

Figure S21. HRESIMS spectrum of saliniquinone H (**2**).

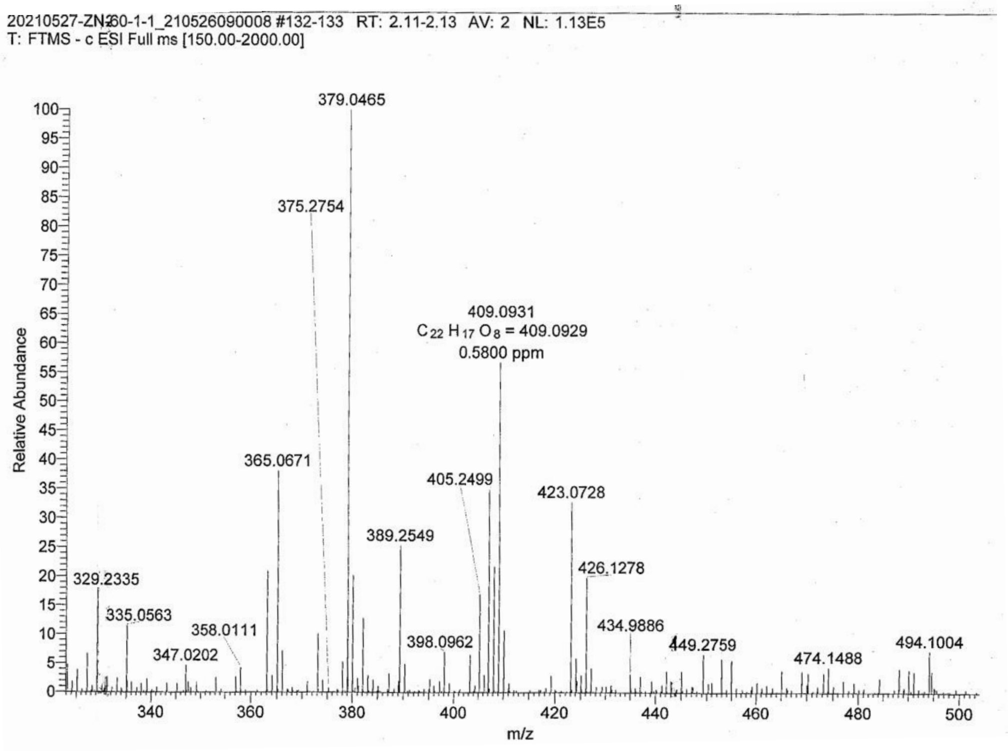

Figure S22. IR spectrum of saliniquinone H (**2**).

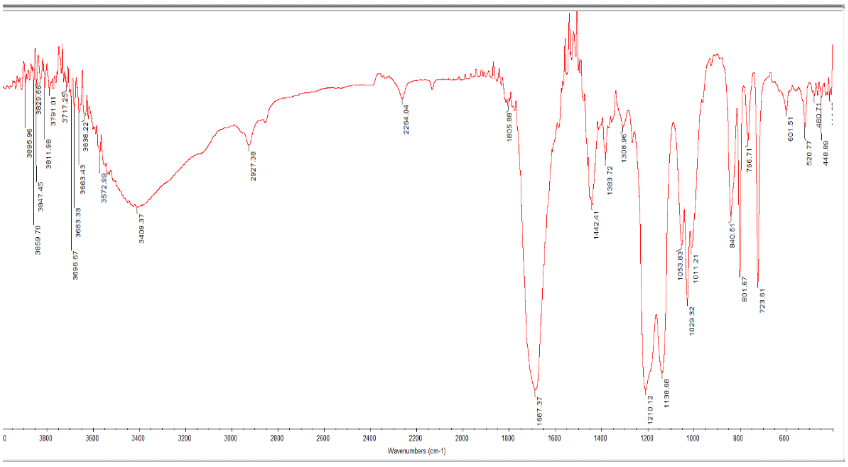

Figure S23. ^{1}H NMR (400 MHz, DMSO) spectrum of saliniquinone I (**3**).

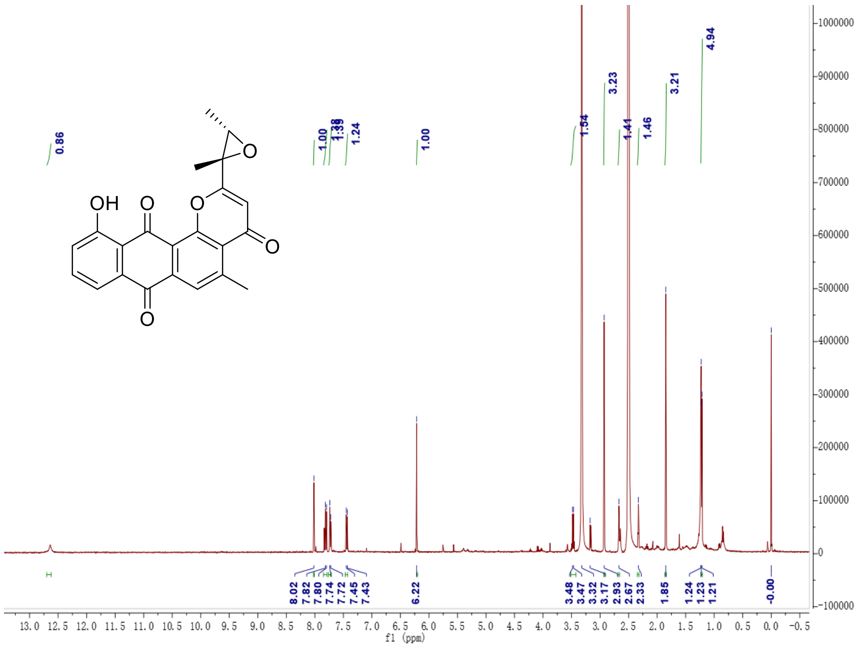

Figure S24. ^{13}C NMR (150 MHz, DMSO) spectrum of saliniquinone I (**3**).

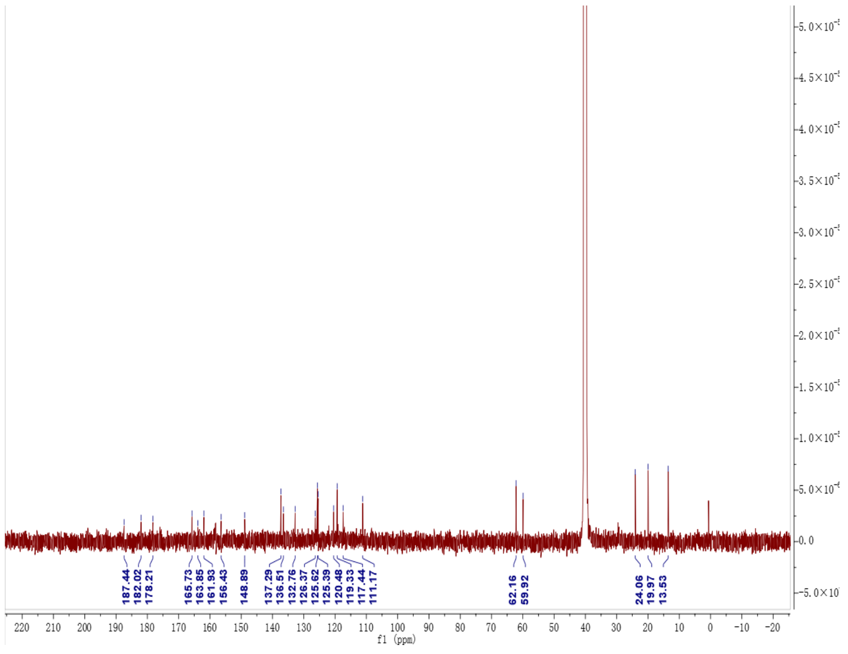

Figure S25. HSQC spectrum of saliniquinone I (**3**).

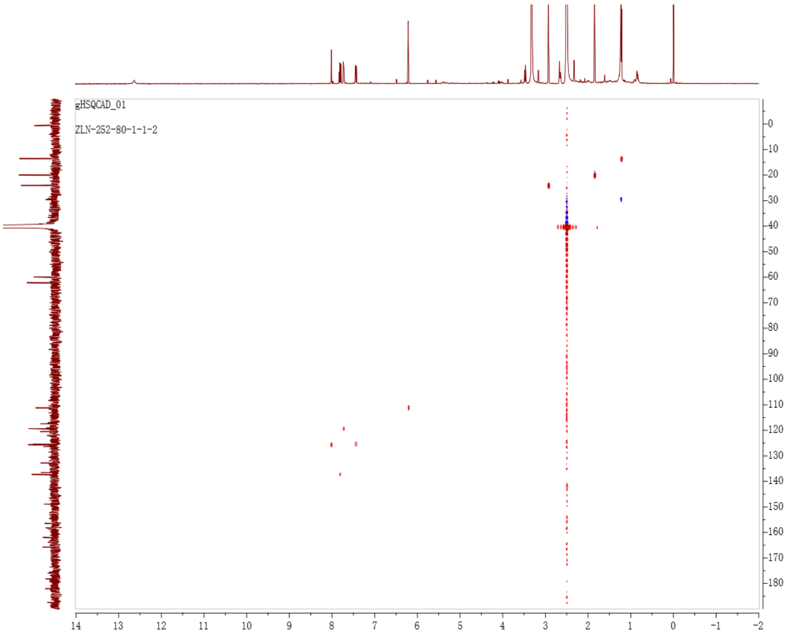

Figure S26. ^{1}H-^{13}C HMBC spectrum of saliniquinone I (**3**).

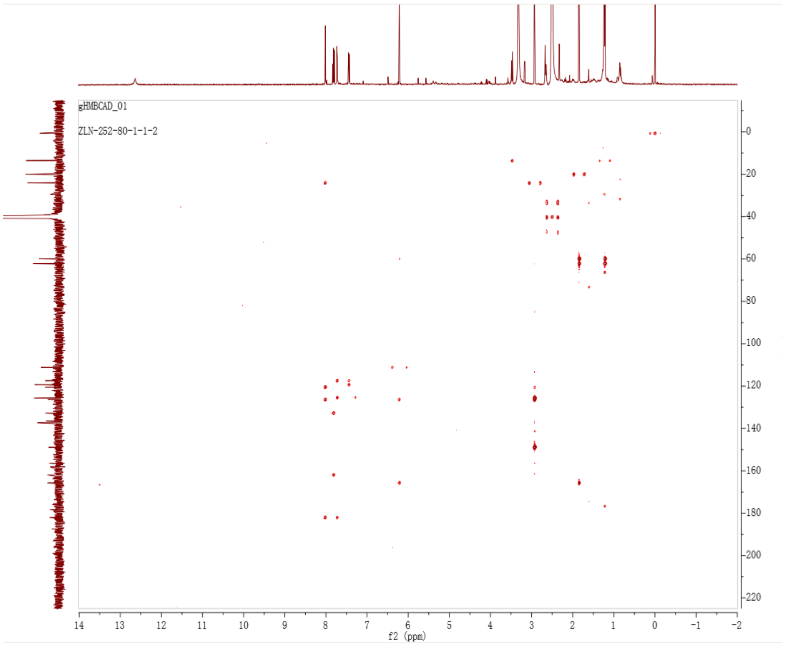

Figure S27. ^{1}H-^{1}H COSY spectrum of saliniquinone I (**3**).

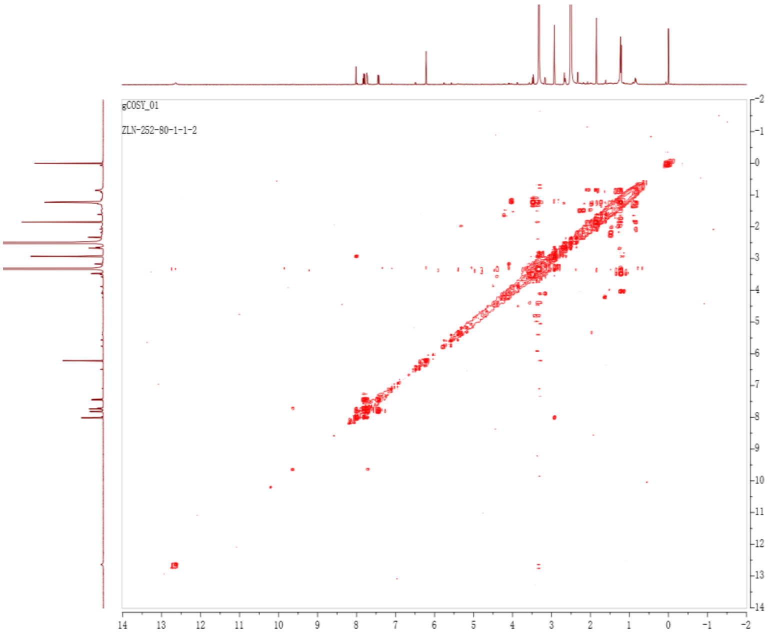

Figure S28. NOESY spectrum of saliniquinone I (**3**).

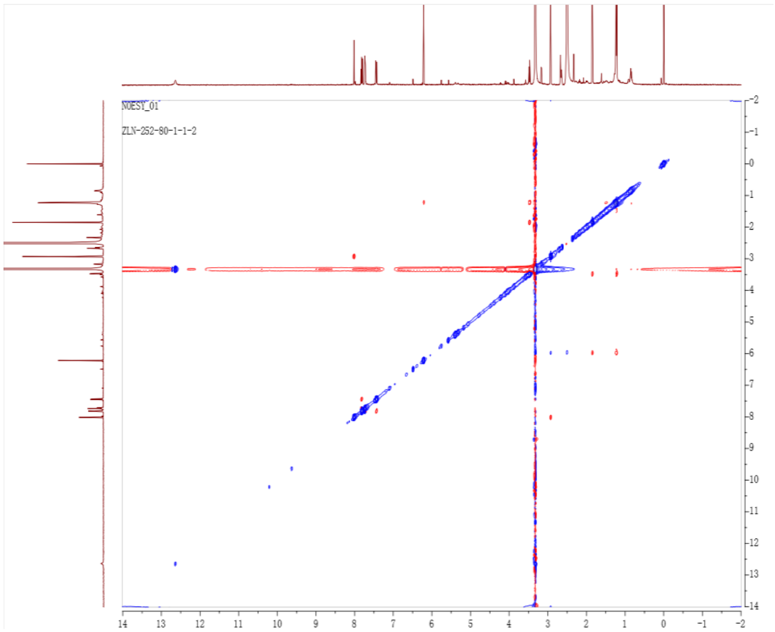

Figure S29. HRESIMR spectrum of saliniquinone I (**3**).

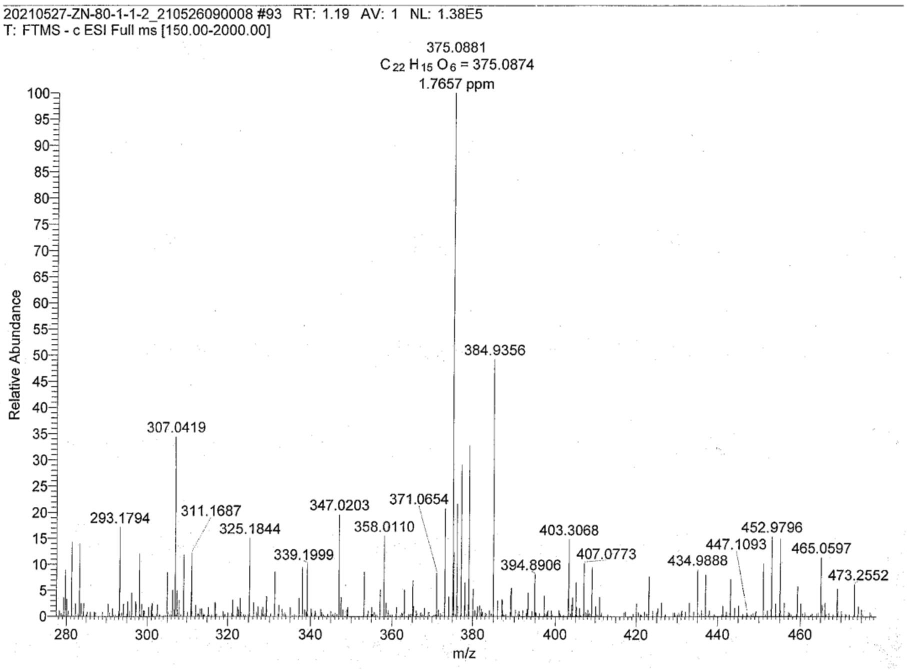

Figure S30. IR spectrum of saliniquinone I (**3**).

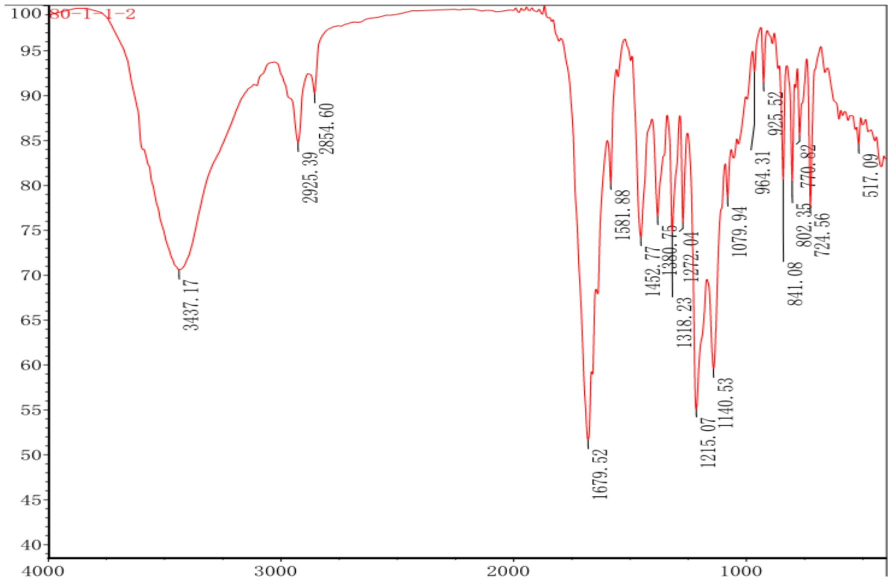

Figure S31. ^{1}H NMR (600 MHz, DMSO) spectrum of heraclemycin E (**4**).

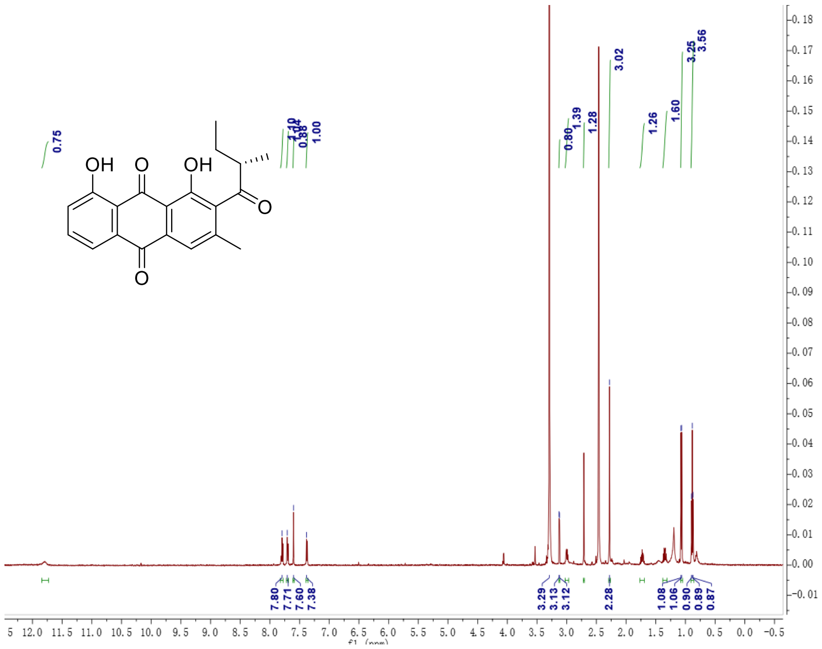

Figure S32. ^{13}C NMR (150 MHz, DMSO) spectrum of heraclemycin E (**4**).

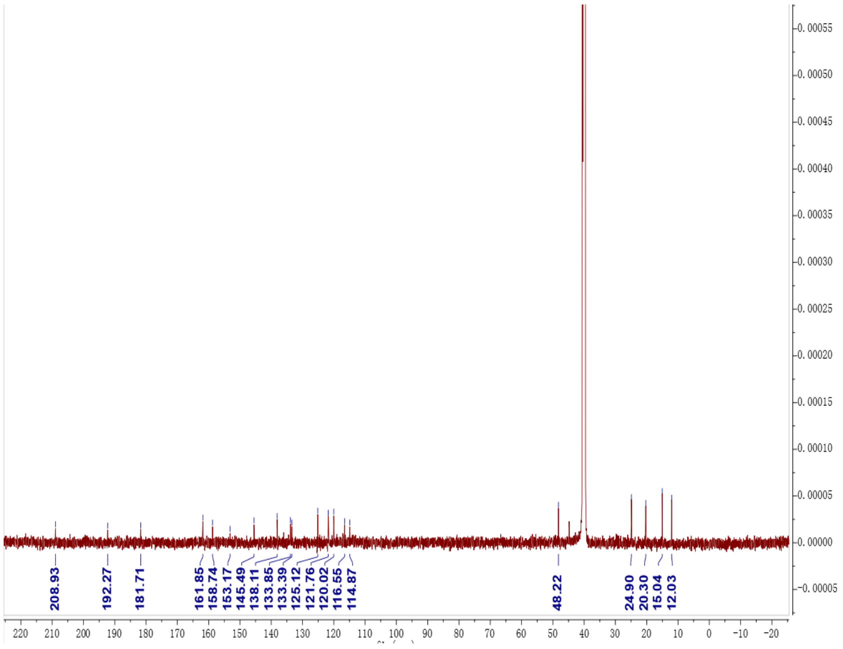

Figure S33. HSQC spectrum of heraclemycin E (**4**).

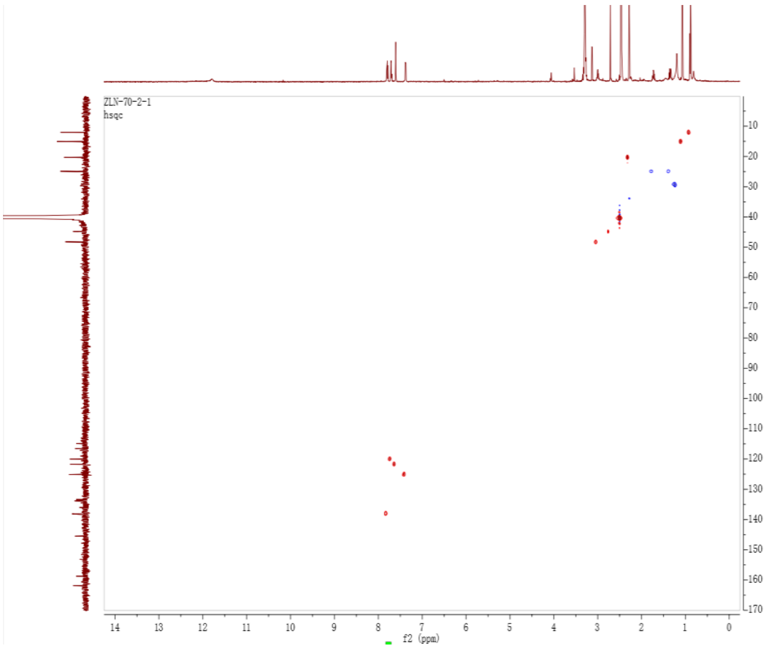

Figure S34. ^{1}H-^{13}C HMBC spectrum of heraclemycin E (**4**).

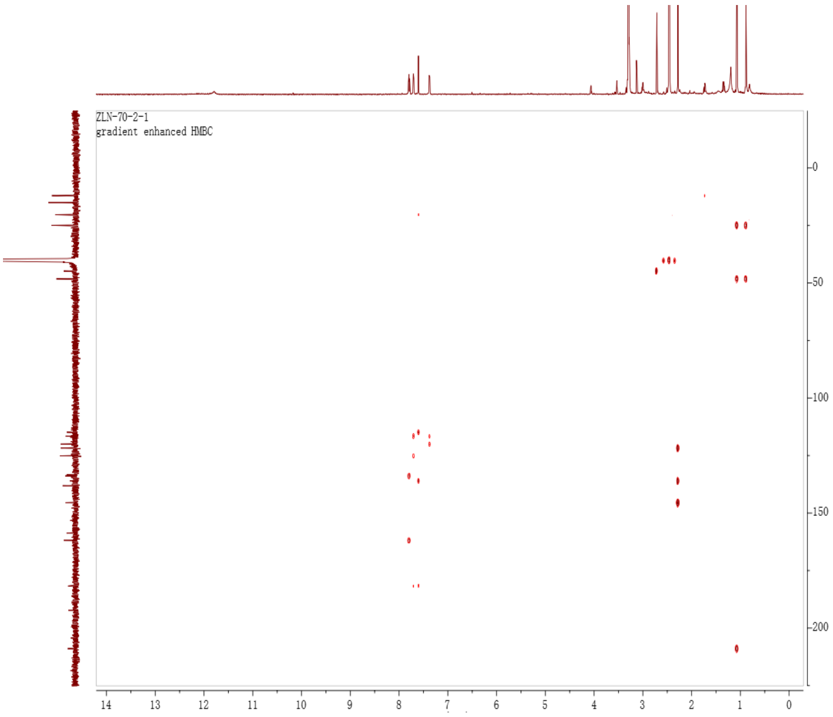

Figure S35. ^{1}H-^{1}H COSY spectrum of heraclemycin E (**4**).

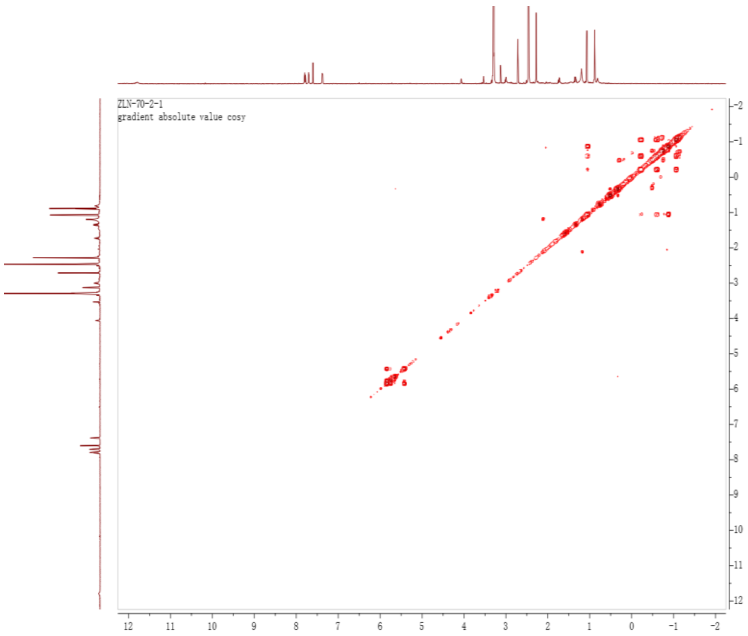

Figure S36. NOESY spectrum of heraclemycin E (**4**).

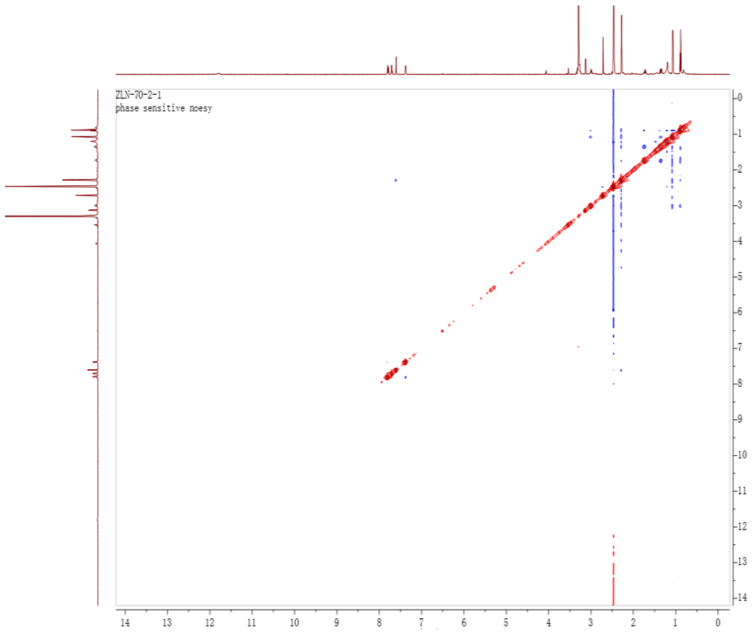

Figure S37. HRESIMS spectrum of heraclemycin E (**4**).

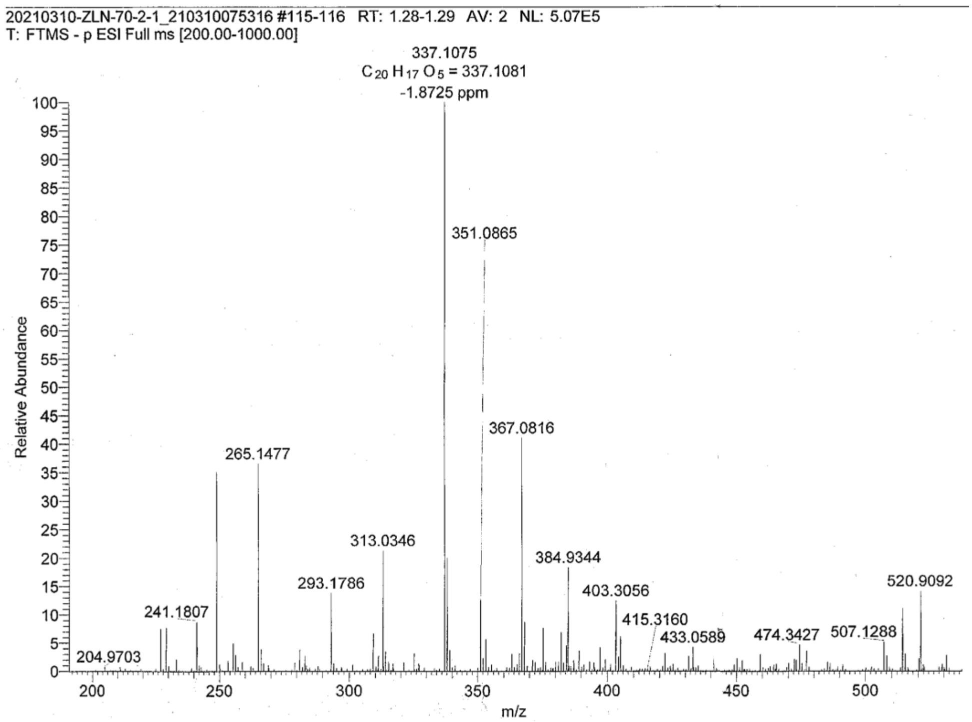

Figure S38. IR spectrum of heraclemycin E (**4**).

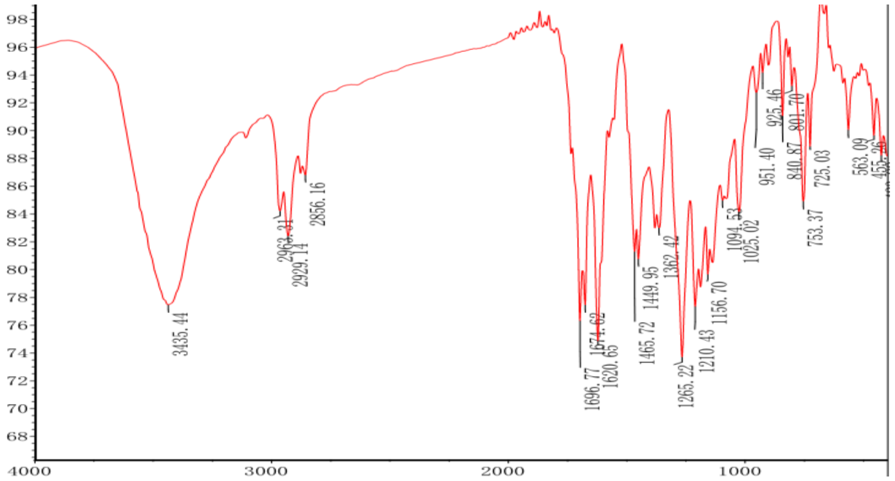

Figure S39. Cartesian coordinates of the low-energy reoptimized conformers of **2a** calculated at B3LYP/6-31+G(d) level of theory.

Conformer A		Standard Orientation (Ångstroms)		
I	Atom	X	Y	Z
1	C	-3.54319	-2.35394	0.01547
2	C	-4.94374	-2.48386	0.06719
3	C	-5.75142	-1.35631	0.09327
4	C	-5.18513	-0.07359	0.06869
5	C	-3.80157	0.07004	0.01665
6	C	-2.95014	-1.06403	-0.01232
7	C	-3.21950	1.43247	-0.00650
8	C	-1.72729	1.56179	-0.06149
9	C	-0.89256	0.43322	-0.10005
10	C	-1.48582	-0.93324	-0.06871
11	C	-1.19150	2.85432	-0.07957
12	C	0.17676	3.08967	-0.12148
13	C	1.05891	1.97295	-0.15370
14	C	0.50577	0.67249	-0.16400
15	O	-0.77133	-1.95449	-0.08531
16	O	-3.92673	2.43859	0.01707
17	C	2.53805	2.10383	-0.19133
18	C	3.27880	0.87388	-0.33764
19	C	2.64779	-0.32112	-0.35096
20	O	1.30411	-0.42433	-0.24721
21	O	3.12531	3.20151	-0.09963
22	C	3.29402	-1.68275	-0.49803
23	C	0.64453	4.53740	-0.16389
24	O	1.38628	4.93382	0.98650
25	O	-2.81850	-3.48728	-0.00642
26	C	2.96695	-2.58628	0.73344
27	O	4.70161	-1.46001	-0.55884
28	C	2.80482	-2.34234	-1.80464
29	C	3.26745	-1.94426	2.08526
30	O	3.78216	-3.75441	0.52248
31	H	-5.36727	-3.48331	0.08619
32	H	-6.83075	-1.46951	0.13338
33	H	-5.81031	0.81184	0.09037
34	H	-1.88347	3.68906	-0.05713
35	H	4.35766	0.91606	-0.40778

36	H	1.23133	4.70800	-1.07596
37	H	-0.23525	5.18365	-0.20248
38	H	2.25257	4.48717	0.87931
39	H	-1.86201	-3.21418	-0.04049
40	H	1.90838	-2.86575	0.67799
41	H	5.11447	-2.32546	-0.38260
42	H	3.06620	-1.70911	-2.65837
43	H	3.29198	-3.31394	-1.92423
44	H	1.72121	-2.49175	-1.78970
45	H	3.10264	-2.67373	2.88732
46	H	2.60206	-1.09600	2.27751
47	H	4.30430	-1.59658	2.13696
48	H	3.820947	-4.26588	1.346483

Conformer B		Standard Orientation (Ångstroms)		
I	Atom	X	Y	Z
1	C	-3.54737	-2.3366	-0.22009
2	C	-4.94809	-2.46546	-0.17003
3	C	-5.74842	-1.34663	0.009451
4	C	-5.17457	-0.07415	0.143841
5	C	-3.79081	0.068518	0.09455
6	C	-2.94679	-1.05647	-0.08974
7	C	-3.20073	1.41994	0.239285
8	C	-1.70958	1.550907	0.163822
9	C	-0.88186	0.431759	-0.02237
10	C	-1.48248	-0.92613	-0.1476
11	C	-1.16719	2.834716	0.288231
12	C	0.199476	3.072467	0.218707
13	C	1.073155	1.967559	0.014389
14	C	0.51655	0.672099	-0.08191
15	O	-0.77435	-1.94016	-0.30254
16	O	-3.90067	2.416004	0.415241
17	C	2.548818	2.104278	-0.08735
18	C	3.293643	0.871253	-0.17565
19	C	2.660352	-0.31988	-0.25675
20	O	1.312478	-0.41913	-0.23404
21	O	3.129039	3.209333	-0.10108
22	C	3.309126	-1.67811	-0.42372
23	C	0.678432	4.506016	0.397448
24	O	1.290887	5.054339	-0.76668

25	O	-2.82941	-3.46099	-0.39385
26	C	2.918965	-2.62549	0.755599
27	O	4.718533	-1.45879	-0.40604
28	C	2.883171	-2.28697	-1.77616
29	C	3.153284	-2.03616	2.144022
30	O	3.739924	-3.78892	0.541876
31	H	-5.37773	-3.45699	-0.27457
32	H	-6.82797	-1.45886	0.04635
33	H	-5.79402	0.804224	0.285422
34	H	-1.85261	3.660968	0.441929
35	H	4.374294	0.911756	-0.20983
36	H	-0.18758	5.134645	0.61655
37	H	1.363668	4.561749	1.253288
38	H	2.161663	4.606651	-0.81367
39	H	-1.87184	-3.18924	-0.40377
40	H	1.863598	-2.89764	0.636816
41	H	5.120453	-2.33178	-0.24237
42	H	3.185076	-1.62254	-2.59194
43	H	3.375389	-3.25431	-1.90894
44	H	1.800082	-2.43484	-1.81832
45	H	2.940986	-2.79275	2.908941
46	H	2.485612	-1.18889	2.332736
47	H	4.188683	-1.70077	2.262529
48	H	3.738206	-4.32977	1.347832

Conformer C		Standard Orientation (Ångstroms)		
I	Atom	X	Y	Z
1	C	3.681998	-1.99603	0.0476
2	C	5.088126	-1.98001	-0.02366
3	C	5.77234	-0.77746	-0.11014
4	C	5.07449	0.439566	-0.12609
5	C	3.684989	0.439361	-0.06785
6	C	2.955108	-0.77539	0.01136
7	C	2.964144	1.732957	-0.07153
8	C	1.465292	1.705411	-0.06794
9	C	0.750232	0.495345	-0.04669
10	C	1.488958	-0.78885	0.067075
11	C	0.801896	2.935316	-0.10852
12	C	-0.58336	3.027107	-0.11657
13	C	-1.34478	1.82517	-0.11001

14	C	-0.66722	0.584838	-0.11296
15	O	0.893015	-1.87709	0.222863
16	O	3.561822	2.807304	-0.08892
17	C	-2.82715	1.809447	-0.11333
18	C	-3.44447	0.512587	-0.26445
19	C	-2.71199	-0.62215	-0.30746
20	O	-1.35606	-0.58657	-0.20159
21	O	-3.52048	2.838871	0.004915
22	C	-3.22946	-2.04721	-0.49974
23	C	-1.20151	4.416439	-0.17599
24	O	-1.92719	4.768137	0.999045
25	O	3.089433	-3.19883	0.144383
26	C	-2.70158	-3.0214	0.614342
27	O	-2.73495	-2.51283	-1.76272
28	C	-4.75648	-2.08835	-0.57133
29	C	-2.79888	-2.48562	2.040988
30	O	-1.3813	-3.46464	0.288615
31	H	5.614013	-2.92947	-0.00333
32	H	6.857107	-0.77902	-0.16039
33	H	5.603133	1.384363	-0.18239
34	H	1.404921	3.836373	-0.12987
35	H	-4.52428	0.479232	-0.32421
36	H	-0.39678	5.148495	-0.27371
37	H	-1.84367	4.496707	-1.06239
38	H	-2.74973	4.238633	0.943168
39	H	2.112623	-3.03858	0.209231
40	H	-3.31411	-3.92424	0.525803
41	H	-1.90626	-2.99419	-1.56064
42	H	-5.12182	-1.48803	-1.40989
43	H	-5.0689	-3.1232	-0.73608
44	H	-5.22377	-1.72748	0.349418
45	H	-2.49994	-3.27008	2.743438
46	H	-3.82469	-2.18759	2.283235
47	H	-2.14447	-1.62094	2.197373
48	H	-0.73181	-2.74224	0.415783

Conformer D		Standard Orientation (Ångstroms)		
I	Atom	X	Y	Z
1	C	-3.21885	-2.71612	-0.09749
2	C	-4.59846	-2.98774	-0.03872

3	C	-5.51312	-1.94926	0.060981
4	C	-5.07678	-0.61754	0.105029
5	C	-3.71476	-0.33419	0.04785
6	C	-2.75658	-1.37432	-0.05531
7	C	-3.2701	1.07943	0.097468
8	C	-1.79949	1.360451	0.031722
9	C	-0.85858	0.320366	-0.07552
10	C	-1.31232	-1.09453	-0.11883
11	C	-1.40022	2.698986	0.080243
12	C	-0.06043	3.067919	0.024865
13	C	0.92419	2.049146	-0.08693
14	C	0.507048	0.700846	-0.13545
15	O	-0.50207	-2.03914	-0.20685
16	O	-4.07581	2.004637	0.189579
17	C	2.378875	2.344551	-0.15436
18	C	3.250204	1.192761	-0.26349
19	C	2.746856	-0.05912	-0.3052
20	O	1.416183	-0.30457	-0.24557
21	O	2.824543	3.503568	-0.11893
22	C	3.533451	-1.34543	-0.45082
23	C	0.303237	4.534939	0.08539
24	O	-0.89092	5.312214	0.200325
25	O	-2.38494	-3.76799	-0.19415
26	C	3.269453	-2.29707	0.759595
27	O	4.912609	-0.97908	-0.46884
28	C	3.152388	-2.03417	-1.7781
29	C	3.457438	-1.64646	2.127662
30	O	4.211276	-3.36861	0.561373
31	H	-4.92124	-4.02378	-0.07369
32	H	-6.57528	-2.17154	0.105082
33	H	-5.78584	0.198883	0.18334
34	H	-2.16158	3.462084	0.163751
35	H	4.320298	1.345697	-0.31212
36	H	0.966269	4.716169	0.939542
37	H	0.861757	4.815458	-0.81541
38	H	-0.6324	6.245963	0.240287
39	H	-1.46174	-3.39555	-0.22456
40	H	2.249372	-2.6881	0.66819
41	H	5.405941	-1.80098	-0.29093
42	H	3.368927	-1.36482	-2.61672
43	H	3.742746	-2.94718	-1.89447
44	H	2.090591	-2.29684	-1.79618
45	H	3.347587	-2.40035	2.916599

46	H	2.698716	-0.87748	2.30719
47	H	4.448439	-1.18962	2.216708
48	H	4.289592	-3.87685	1.38437

Conformer E		Standard Orientation (Ångstroms)		
I	Atom	X	Y	Z
1	C	3.688708	-1.98269	-0.14595
2	C	5.094391	-1.95292	-0.22172
3	C	5.77326	-0.7448	-0.18629
4	C	5.070301	0.464048	-0.07217
5	C	3.681137	0.451327	-0.00721
6	C	2.956511	-0.76827	-0.05143
7	C	2.954893	1.734301	0.129679
8	C	1.45595	1.699229	0.137594
9	C	0.744847	0.491097	0.032824
10	C	1.49051	-0.79439	0.009295
11	C	0.788502	2.922859	0.241736
12	C	-0.59694	3.012582	0.228586
13	C	-1.35379	1.818166	0.069642
14	C	-0.67334	0.582002	-0.02139
15	O	0.900833	-1.8963	0.048278
16	O	3.547662	2.807324	0.224526
17	C	-2.83502	1.805266	0.010954
18	C	-3.45377	0.504713	-0.09073
19	C	-2.72027	-0.62421	-0.20568
20	O	-1.36071	-0.58392	-0.17579
21	O	-3.52778	2.841876	0.033941
22	C	-3.24452	-2.04248	-0.42983
23	C	-1.21575	4.391821	0.408342
24	O	-1.92742	4.857029	-0.73493
25	O	3.101844	-3.19184	-0.17262
26	C	-2.658	-3.06021	0.613473
27	O	-2.81793	-2.45344	-1.73556
28	C	-4.77331	-2.08647	-0.42029
29	C	-2.67661	-2.58069	2.063156
30	O	-1.35893	-3.49038	0.198484
31	H	5.623967	-2.89702	-0.30325
32	H	6.857708	-0.73589	-0.24192
33	H	5.595108	1.411791	-0.03215
34	H	1.388629	3.820903	0.33801

35	H	-4.53481	0.469405	-0.11427
36	H	-1.86715	4.389471	1.291776
37	H	-0.41111	5.109161	0.584145
38	H	-2.75248	4.328585	-0.73936
39	H	2.124535	-3.04337	-0.08927
40	H	-3.27629	-3.95895	0.522977
41	H	-1.98259	-2.94569	-1.59803
42	H	-5.18457	-1.45837	-1.21605
43	H	-5.0927	-3.11589	-0.60471
44	H	-5.19099	-1.76029	0.536639
45	H	-2.34426	-3.3926	2.717779
46	H	-3.68662	-2.28913	2.370814
47	H	-2.01101	-1.72429	2.217878
48	H	-0.70208	-2.77236	0.312765

Figure S40. Cartesian coordinates of the low-energy reoptimized conformers of **2b** calculated at B3LYP/6-31+G(d) level of theory.

Conformer A		Standard Orientation (Ångstroms)		
I	Atom	X	Y	Z
1	C	-3.53201	-2.34761	0.030493
2	C	-4.93059	-2.49422	0.068608
3	C	-5.74927	-1.37001	0.068489
4	C	-5.18796	-0.08165	0.031024
5	C	-3.80155	0.074877	-0.0079
6	C	-2.94137	-1.05701	-0.01106
7	C	-3.22416	1.431041	-0.04156
8	C	-1.7397	1.571834	-0.0781
9	C	-0.89491	0.44368	-0.09738
10	C	-1.48235	-0.91581	-0.05303
11	C	-1.20884	2.868427	-0.0988
12	C	0.162688	3.112762	-0.12255
13	C	1.052666	1.999267	-0.13285
14	C	0.499841	0.695841	-0.14957
15	O	-0.75488	-1.96145	-0.04044
16	O	-3.95259	2.454874	-0.04113
17	C	2.52795	2.132642	-0.14056
18	C	3.296261	0.92021	-0.26182
19	C	2.695575	-0.29243	-0.29799
20	O	1.326339	-0.4069	-0.22971

21	O	3.114182	3.260842	-0.03819
22	C	3.365083	-1.65161	-0.4644
23	C	0.62083	4.559023	-0.16786
24	O	1.367204	4.968887	1.022292
25	O	-2.78335	-3.49636	0.035216
26	C	2.832143	-2.63524	0.611147
27	O	4.801858	-1.47578	-0.28275
28	C	3.153956	-2.15625	-1.89814
29	C	3.336631	-4.06835	0.478545
30	O	3.316707	-2.02341	1.869898
31	H	-5.34254	-3.49605	0.098554
32	H	-6.82657	-1.48826	0.098596
33	H	-5.81924	0.798285	0.033486
34	H	-1.90392	3.699226	-0.09293
35	H	4.373556	0.990702	-0.30682
36	H	1.231173	4.734824	-1.06102
37	H	-0.25229	5.210244	-0.20614
38	H	2.257321	4.545192	0.938454
39	H	-1.81196	-3.24514	0.010281
40	H	1.738972	-2.60509	0.600565
41	H	4.982873	-1.43413	0.684156
42	H	3.521046	-1.41098	-2.60951
43	H	3.71175	-3.083	-2.04886
44	H	2.09329	-2.33854	-2.09373
45	H	3.015383	-4.65297	1.348446
46	H	2.917519	-4.55154	-0.40969
47	H	4.428713	-4.10296	0.418791
48	H	3.095653	-2.57204	2.647324

Conformer B		Standard Orientation (Ångstroms)		
I	Atom	X	Y	Z
1	C	-3.5368	-2.33653	-0.18526
2	C	-4.93615	-2.48012	-0.17107
3	C	-5.7502	-1.35981	-0.04289
4	C	-5.18349	-0.07866	0.074518
5	C	-3.79634	0.075069	0.058381
6	C	-2.94081	-1.05242	-0.07516
7	C	-3.21323	1.422949	0.187633
8	C	-1.72872	1.562923	0.148528
9	C	-0.88839	0.441362	-0.00365

10	C	-1.48118	-0.9132	-0.0998
11	C	-1.19299	2.851691	0.271328
12	C	0.178104	3.096265	0.233767
13	C	1.061819	1.993239	0.049858
14	C	0.506455	0.69453	-0.05038
15	O	-0.75793	-1.95687	-0.20035
16	O	-3.93696	2.440849	0.325099
17	C	2.534821	2.131777	-0.02434
18	C	3.304742	0.917364	-0.10903
19	C	2.702793	-0.29157	-0.20281
20	O	1.33147	-0.40267	-0.19918
21	O	3.116717	3.266738	-0.02562
22	C	3.376615	-1.64494	-0.39805
23	C	0.643965	4.528819	0.418416
24	O	1.306456	5.080982	-0.76404
25	O	-2.79258	-3.4814	-0.3098
26	C	2.804556	-2.6686	0.618612
27	O	4.805634	-1.47848	-0.15551
28	C	3.219006	-2.09405	-1.8569
29	C	3.311113	-4.09676	0.446296
30	O	3.245095	-2.10783	1.916714
31	H	-5.35243	-3.47659	-0.26057
32	H	-6.82812	-1.47577	-0.03186
33	H	-5.81107	0.797824	0.178232
34	H	-1.88353	3.67617	0.400886
35	H	4.382834	0.986274	-0.13174
36	H	-0.22201	5.166794	0.594437
37	H	1.315896	4.60067	1.281159
38	H	2.200278	4.658637	-0.79219
39	H	-1.82016	-3.23298	-0.29994
40	H	1.712479	-2.63488	0.570222
41	H	4.949371	-1.47442	0.818502
42	H	3.6081	-1.32089	-2.52551
43	H	3.785657	-3.01273	-2.02319
44	H	2.166901	-2.27181	-2.09744
45	H	2.959628	-4.71519	1.28031
46	H	2.921609	-4.54216	-0.47456
47	H	4.404532	-4.13214	0.422181
48	H	2.992637	-2.6835	2.664511

Conformer C		Standard Orientation (Ångstroms)		
I	Atom	X	Y	Z
1	C	-3.52047	-2.39446	-0.05092
2	C	-4.9148	-2.5599	0.034921
3	C	-5.7451	-1.44685	0.112962
4	C	-5.19986	-0.15118	0.106214
5	C	-3.81736	0.023712	0.024948
6	C	-2.94552	-1.09615	-0.05334
7	C	-3.25604	1.386627	0.016262
8	C	-1.77419	1.546529	-0.05196
9	C	-0.91572	0.430466	-0.11624
10	C	-1.48993	-0.93564	-0.13722
11	C	-1.26139	2.850506	-0.05302
12	C	0.105502	3.113733	-0.101
13	C	1.010144	2.012609	-0.13897
14	C	0.476651	0.700647	-0.16212
15	O	-0.75383	-1.97018	-0.22831
16	O	-3.99514	2.401426	0.068288
17	C	2.481866	2.169445	-0.17216
18	C	3.266114	0.969658	-0.31711
19	C	2.684803	-0.2531	-0.33413
20	O	1.318738	-0.39142	-0.2393
21	O	3.053994	3.304482	-0.06936
22	C	3.393569	-1.58819	-0.43453
23	C	0.54303	4.566604	-0.13736
24	O	1.302482	4.974348	1.045402
25	O	-2.75988	-3.53267	-0.12997
26	C	3.568647	-2.22446	0.98549
27	O	4.741818	-1.26324	-0.9118
28	C	2.701938	-2.5264	-1.43485
29	C	2.323525	-2.83746	1.610934
30	O	4.607997	-3.25433	0.764669
31	H	-5.31461	-3.56708	0.036493
32	H	-6.81916	-1.57969	0.177815
33	H	-5.84042	0.720154	0.163657
34	H	-1.96732	3.671272	-0.01609
35	H	4.340448	1.051789	-0.39016
36	H	1.136517	4.760616	-1.03801
37	H	-0.33952	5.205863	-0.15443
38	H	2.197953	4.567144	0.94178
39	H	-1.79379	-3.26755	-0.19236

40	H	3.991894	-1.4525	1.639875
41	H	5.324117	-2.0044	-0.62584
42	H	2.723096	-2.07316	-2.4306
43	H	3.239886	-3.47749	-1.46965
44	H	1.662201	-2.70639	-1.15365
45	H	2.560653	-3.20796	2.614297
46	H	1.521327	-2.0996	1.700454
47	H	1.956799	-3.67417	1.009899
48	H	4.998036	-3.55466	1.608723

Conformer D		Standard Orientation (Ångstroms)		
I	Atom	X	Y	Z
1	C	-3.52561	-2.37386	-0.28117
2	C	-4.9203	-2.54189	-0.20678
3	C	-5.74326	-1.44376	0.019709
4	C	-5.19036	-0.16076	0.175292
5	C	-3.8076	0.016487	0.104509
6	C	-2.94316	-1.08844	-0.12406
7	C	-3.2386	1.366891	0.263413
8	C	-1.75801	1.53066	0.180571
9	C	-0.90654	0.427144	-0.02922
10	C	-1.48766	-0.92519	-0.20108
11	C	-1.23862	2.823947	0.325768
12	C	0.126502	3.090614	0.255723
13	C	1.022472	2.003736	0.03667
14	C	0.486012	0.697421	-0.07172
15	O	-0.75787	-1.94433	-0.42339
16	O	-3.97042	2.368044	0.465792
17	C	2.489773	2.167944	-0.06952
18	C	3.277967	0.966282	-0.17299
19	C	2.695209	-0.25374	-0.2457
20	O	1.325469	-0.38941	-0.21909
21	O	3.053496	3.311675	-0.07932
22	C	3.403278	-1.58744	-0.36609
23	C	0.575893	4.527232	0.449086
24	O	1.192894	5.106711	-0.74497
25	O	-2.77178	-3.49647	-0.50853
26	C	3.539025	-2.26245	1.039751
27	O	4.765811	-1.25371	-0.79605
28	C	2.738008	-2.49575	-1.41079

29	C	2.272115	-2.8703	1.625258
30	O	4.56665	-3.30246	0.813729
31	H	-5.32621	-3.53898	-0.33053
32	H	-6.81763	-1.57838	0.075236
33	H	-5.82526	0.69923	0.34907
34	H	-1.93786	3.633726	0.495213
35	H	4.353731	1.048575	-0.22079
36	H	-0.29389	5.148853	0.661022
37	H	1.273213	4.59701	1.291504
38	H	2.092459	4.70089	-0.80606
39	H	-1.8044	-3.22984	-0.54188
40	H	3.960512	-1.51394	1.721962
41	H	5.336166	-2.0092	-0.52454
42	H	2.792477	-2.01651	-2.39297
43	H	3.272016	-3.44863	-1.45385
44	H	1.688935	-2.6766	-1.16813
45	H	2.488127	-3.28467	2.616113
46	H	1.486934	-2.11703	1.737132
47	H	1.89277	-3.67423	0.988503
48	H	4.934739	-3.62936	1.657822

Conformer E		Standard Orientation (Ångstroms)		
I	Atom	X	Y	Z
1	C	-3.49269	-2.50103	0.141955
2	C	-4.88432	-2.70643	0.103781
3	C	-5.74402	-1.62264	-0.03069
4	C	-5.23283	-0.31585	-0.13033
5	C	-3.85548	-0.09898	-0.09228
6	C	-2.95216	-1.191	0.047006
7	C	-3.33639	1.278596	-0.20126
8	C	-1.86111	1.48511	-0.14784
9	C	-0.98125	0.39549	0.000677
10	C	-1.50476	-0.98588	0.09173
11	C	-1.36765	2.793467	-0.25272
12	C	-0.00412	3.08728	-0.20576
13	C	0.917498	2.013805	-0.03297
14	C	0.397198	0.704528	0.051907
15	O	-0.73099	-1.99776	0.20742
16	O	-4.10508	2.262887	-0.33451
17	C	2.389043	2.180227	0.044802

18	C	3.18948	0.978499	0.126354
19	C	2.613381	-0.24475	0.202818
20	O	1.248105	-0.36545	0.187284
21	O	2.946941	3.325354	0.049911
22	C	3.254322	-1.61613	0.401731
23	C	0.416961	4.53546	-0.37039
24	O	1.078705	5.084563	0.813346
25	O	-2.70282	-3.61235	0.274105
26	C	4.392186	-1.83534	-0.63355
27	O	2.265987	-2.66027	0.102621
28	C	3.721258	-1.75511	1.863074
29	C	5.177417	-3.13077	-0.43318
30	O	3.79258	-1.7972	-1.97342
31	H	-5.25725	-3.72085	0.180662
32	H	-6.81543	-1.78554	-0.05989
33	H	-5.89819	0.531913	-0.23731
34	H	-2.08519	3.595632	-0.37681
35	H	4.264537	1.096296	0.151186
36	H	1.07574	4.640261	-1.24016
37	H	-0.46813	5.152051	-0.52561
38	H	1.986187	4.692373	0.824755
39	H	-1.74176	-3.32784	0.284648
40	H	5.071758	-0.97994	-0.61244
41	H	1.338246	-2.34096	0.217509
42	H	2.89519	-1.52939	2.544639
43	H	4.047498	-2.78167	2.043599
44	H	4.549417	-1.07246	2.080986
45	H	5.876342	-3.25209	-1.26633
46	H	5.758963	-3.10774	0.494042
47	H	4.508421	-3.99672	-0.40678
48	H	3.034345	-2.4229	-1.97998
